# Single-cell metabolic profiling reveals subgroups of primary human hepatocytes with heterogeneous responses to drug challenge

**DOI:** 10.1186/s13059-023-03075-9

**Published:** 2023-10-17

**Authors:** Eva Sanchez-Quant, Maria Lucia Richter, Maria Colomé-Tatché, Celia Pilar Martinez-Jimenez

**Affiliations:** 1 Helmholtz Pioneer Campus (HPC), Helmholtz Zentrum München, 85764 Neuherberg, Germany; 2 Institute of Computational Biology, Helmholtz Zentrum München, German Research Center for Environmental Health, 85764 Neuherberg, Germany; 3 TUM School of Life Sciences Weihenstephan, Technical University of Munich (TUM), 85354 Freising, Germany; 4 Biomedical Center (BMC), Physiological Chemistry, Faculty of Medicine, Ludwig Maximilian University of Munich (LMU), 82152 Munich, Germany; 5 TUM School of Medicine, Technical University of Munich, Munich (TUM), 80333 Munich, Germany

**Keywords:** Primary human hepatocytes, Drug metabolism, Single-cell transcriptomics, Hepatic steatosis, NAFLD, DILI, Lipid metabolism, Cytochrome P450, Liver

## Abstract

**Background:**

Xenobiotics are primarily metabolized by hepatocytes in the liver, and primary human hepatocytes are the gold standard model for the assessment of drug efficacy, safety, and toxicity in the early phases of drug development. Recent advances in single-cell genomics demonstrate liver zonation and ploidy as main drivers of cellular heterogeneity. However, little is known about the impact of hepatocyte specialization on liver function upon metabolic challenge, including hepatic metabolism, detoxification, and protein synthesis.

**Results:**

Here, we investigate the metabolic capacity of individual human hepatocytes in vitro. We assess how chronic accumulation of lipids enhances cellular heterogeneity and impairs the metabolisms of drugs. Using a phenotyping five-probe cocktail, we identify four functional subgroups of hepatocytes responding differently to drug challenge and fatty acid accumulation. These four subgroups display differential gene expression profiles upon cocktail treatment and xenobiotic metabolism-related specialization. Notably, intracellular fat accumulation leads to increased transcriptional variability and diminishes the drug-related metabolic capacity of hepatocytes.

**Conclusions:**

Our results demonstrate that, upon a metabolic challenge such as exposure to drugs or intracellular fat accumulation, hepatocyte subgroups display different and heterogeneous transcriptional responses.

**Supplementary Information:**

The online version contains supplementary material available at 10.1186/s13059-023-03075-9.

## Background

Single-cell sequencing technologies have revealed a wealth of information regarding cellular heterogeneity in health and disease in multiple tissues [1–8]. In particular, single-cell RNA sequencing enables the identification of groups of cells in a population with similar transcriptomic profiles, which is generally associated with similar functionality [9, 10]. In the liver, single-cell transcriptomic analyses have shown novel cell types and states involved in the development and progression of liver disease [1, 7, 11], as well as in the transcriptomic responses to xenobiotics [12]. However, drug toxicity and safety are generally assessed in primary human hepatocytes (PHH) as the gold standard model to study the drug metabolism in humans, which are widely considered a seemingly homogeneous population of cells [13, 14]. Whether all hepatocytes share the same functional molecular phenotype in vitro remains unknown.

In the liver, drug and xenobiotic metabolism is mainly performed by hepatocytes, the major and predominant cell type of the parenchyma [15–17]. The hepatic metabolism of drugs occurs in three phases. In phase I, oxidation, hydrolysis, reduction, and cyclization reactions are catabolized mainly by the cytochrome P450 (CYP450) superfamily of monooxygenase enzymes. The main members are the isoforms CYP1A2, 2C9, 2C19, 2D6, and 3A4, which account for the metabolism of around 70–80% of the clinically available drugs [18–20]. The expression of these cytochromes is induced by the presence of their substrate compounds, which are indirectly used as a measure of liver metabolic capacity [10, 21]. These substrates act as hepatocyte probes when administered to phenotype and monitor the cytochrome enzymatic activity, formally known as the “phenotyping cocktail approach” [22–28]. Phase II comprises conjugation reactions catabolized mainly by transferase enzymes and are purposed to hydrophilize compounds for their elimination [29]. During phase III of xenobiotic metabolism, conjugated compounds are excreted out of the cell through active transmembrane transporters [30]. The cytochrome superfamily consists of nearly 60 members in humans (Human Genome Project 2013), which may be expressed differently in individual cells when challenged by xenobiotics, leading to cellular heterogeneity that remains concealed in a bulk analysis.

An additional potential source of cellular heterogeneity is the intracellular accumulation of triglycerides in hepatocytes, known as hepatic steatosis [31, 32]. This is a hallmark of non-alcoholic fatty liver disease (NAFLD) and is generally associated with metabolic dysfunction, inflammation, and risk of fibrosis [33, 34]. In culture, intracytoplasmic fat accumulation can be modeled by incubating primary human hepatocytes with free fatty acids (FFA) to mimic benign chronic steatosis [35, 36]. However, lipid accumulation is heterogenous in regards to the number and size of lipid droplets [37] and it still remains unclear whether all cells respond to lipid accumulation in a coordinated manner.

Moreover, the co-administration of five or more drugs (polypharmacy) is associated with a higher incidence of adverse drug reactions (ADR) and drug-induced liver injury (DILI) [38–40]. Importantly, a higher incidence of DILI has been reported in patients suffering from NAFLD [41–43]. Therefore, the precise molecular pathways commonly dysregulated between chronic accumulation of lipid and drug metabolism remain unexplored at cellular resolution.

Here, we report concealed cellular heterogeneity in primary human hepatocytes which are classically considered a seemingly homogenous population of cells and a “gold standard” for in vitro hepatic assays. The comparison between pseudobulk and single-cell transcriptomics reveals four distinct hepatocyte subgroups that can be found also in fourteen human donors from publicly available data sets. We present evidence that chronic accumulation of lipids and xenobiotics leads to transcriptional variability and the impairment of multiple metabolic pathways in a subgroup-specific manner. Our results suggest that liver steatosis combined with multiple drug intake differentially affects the transcriptional profiles of individual cells decreasing the expression of key cytochrome P450 genes.

## Results

### Single-cell RNA-seq reveals four major subgroups of hepatocytes showing cellular heterogeneity and functional specialization in primary human hepatocytes

Liver function is compartmentalized along the liver lobule, and the maintenance of liver function is regulated by liver-enriched transcription factors [44, 45]. Primary human hepatocytes are considered the gold standard model to predict drug responses in humans, but they are characterized by phenotypic instability in culture and rapid loss of the hepatic phenotype [13, 46]. Therefore, we aimed to investigate the level of cellular heterogeneity that remains in a seemingly homogeneous population of primary human hepatocytes (PHHs), and how this heterogeneity might affect pharmaco-toxicological studies [47].

**Fig. 1:**
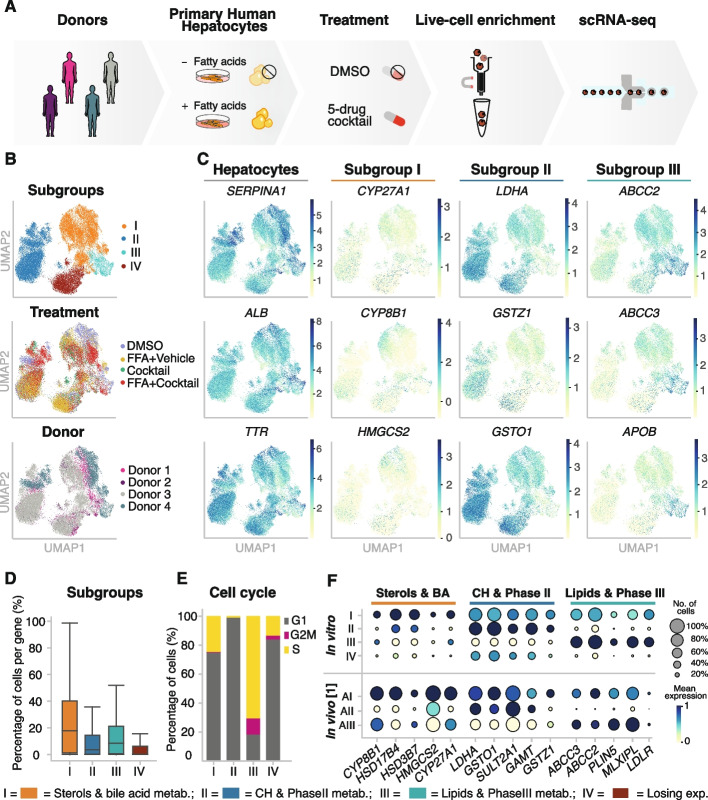
Transcriptomic profiling reveals four subgroups of PHHs independently of donor and treatment condition. **A** Overview of experimental design. Purified cryopreserved PHHs from four human donors were plated, incubated with or without free fatty acids to model hepatic fat accumulation and with or without a phenotypic 5-drug cocktail (Sanofi-Aventis). **B** UMAP colored by subgroup, treatment, and donor showing that the annotated subgroups are found throughout all donors and conditions. **C** UMAP colored by expression levels of—from left to right: (i) key hepatocyte marker genes, (ii) bile acid and sterol metabolism; (iii) carbohydrate and phase II metabolism, (iv) lipid and phase III metabolism marker genes in four subgroups of hepatocytes. **D** Boxplot showing the percentage of cells in which a given gene is expressed, colored by the identified subgroups. **E** Bar plot showing the percentage of cells per subgroup assigned to phases G1, S, and G2M by performing cell cycle analysis using *cyclone.* **F** Dot plot highlighting marker gene expression in four subgroups of hepatocytes identified in vitro (top) and in vivo [1]. Subgroup marker gene expression was grouped by aggregated *Louvain* clusters (dot size: fraction of cells in the group; color scale: mean expression in the group)

Cryopreserved and metabolically competent PHHs from four donors were used as in vitro model to characterize the metabolic profile of individual hepatocytes in response to a drug challenge and chronic accumulation of fat (Fig. 1A, “Methods”). The hepatocytes were plated for 6 h and incubated for 66 h with either vehicle (DMSO) or a five-drug cocktail. Chronic accumulation of fat was achieved by incubation with free fatty acids (FFA) corresponding to a 200 µM mixture of a 2:1 ratio of oleic acid to palmitic acid for 72 h. In brief, four different conditions were studied: (i) vehicle (DMSO 0.5% v/v); (ii) Cocktail (66 h five-drug cocktail incubation); (iii) FFA (2:1 oleic:palmitic acid), and (iv) FFA + Cocktail (combination of FFA incubation and five-drug cocktail) (Fig. 1A). Donors were healthy males between 18 and 57 years, with a body mass index (BMI) classified as normal, non-diabetic, and representing the most common age range commercially available (Additional file 2: Table S1). After isolation of viable cells by a quick and non-harsh dead cell removal step, single-cell RNA-seq was immediately performed using 10x  Genomics (Methods).

A total of 38,232 high-quality hepatocytes with an average of 2550 genes per cell were analyzed. Lenient doublet filtering was performed to account for polyploid hepatocytes (Methods). The variation in the number of cells profiled per donor was attributed in part to sample viability, technical differences in cell capture rates in each single-cell RNA-sequencing (scRNA-seq) run, and sequencing depth (Additional file 1: Fig. S1, Methods).

Across all four donors, *Louvain* clustering led to the detection of heterogeneity separating four subgroups of hepatocytes, independently of the treatment (Fig. 1B, Additional file 1: Fig. S1B and C, Methods). *Harmony* [48] was used to correct for batch effects, and *Louvain* clustering was performed on the combined data set (Additional file 1: Fig. S2A). Overall, hepatocyte purity was confirmed by the expression of the hepatocyte-specific genes *ALB*, *SERPINA1*, and *TTR*, among others (Fig. 1C, and Additional file 3: Table S2). Differential expression analysis was used to annotate the four subgroups of hepatocytes present in all four donors and treatment conditions (Fig. 1B, Additional file 3: Table S2, Methods). Representative marker genes illustrate functional specialization. For instance, *ATF6*, *CYP8B1*, and *HMGCS2* for sterol and bile acid synthesis were up-regulated in subgroup I. Subgroup II was represented by *LDHA*, *GSTZ1*, and *GSTO1*, involved in the carbohydrate and phase II metabolism. Subgroup III was characterized by *ABCC2*, *ABCC3*, and *APOB* over-expression, among other genes (Additional file 1: Fig. S2B) involved in lipid and phase III metabolism (Fig. 1C). Lastly, subgroup IV was characterized by the loss of gene expression, which was 11-fold reduced compared to subgroup I (Fig. 1D, “Methods”). The instability of PHHs has been previously characterized for the loss of expression of liver-specific transcription factors and downstream target genes [49, 50].

Across all conditions, 38.2% of the cells were specialized in the metabolism of bile acids and sterols (subgroup I) [51]; 38.7% of the cells were enriched in genes involved in carbohydrate metabolism and phase II enzymes (subgroup II); 5.4% of the cells were responsible for lipid metabolism and expression of phase III enzymes (subgroup III) (Additional file 1: Fig. S2C); and 17.7% of the cells were losing expression (subgroup IV) (Additional file 1: Fig. S2C). To further investigate the metabolic specialization of the PHH subgroups, we focused our analysis on metabolic marker genes, taken from *metabolicatlas.org* (Methods). We found that clustering analysis using metabolic markers alone led to the identification of the same subgroups of hepatocytes as when using all genes (Additional file 1: Fig. S2E and 2F). These results suggest that differences between subgroups of hepatocytes might be driven by their metabolic function.

Interestingly, in subgroup III, the majority of the cells (73.2%) were assigned to S-phase according to the cell cycle phase classification using the tool *cyclone* (Fig. 1E, Additional file 1: Fig. S2A and S3) [52]. Moreover, subgroup II showed a higher expression level of stress marker genes such as *RSP19*, *PRDX1*, *BAX*, *GSTA1*, *LGALS1*, *MTH1*, and *MTHM* (Additional file 1: Fig. S2D, Additional file 3: Table S2). Likewise, subgroup II showed a low percentage of cells in which a gene is expressed, suggesting that these cells might be prone to lose their characteristic hepatocyte-like expression profile in culture.

To further characterize underlying upstream molecular events, we used the ChIP-X Enrichment Analysis tool, ChEA3 [53] to reconstruct the network of putative transcription factors (TFs) regulating gene expression. We focused our analysis on the untreated cells (i.e., DMSO-treated cells) across all donors, to prevent potential effects due to treatment conditions. Considering the top 500 differentially expressed genes per subgroup, key hepatic TFs, such as *HNF4A* [54, 55] and *MLXIPL* [56] were found among the top 25 predicted TFs. Only in subgroup IV a decrease in the expression levels of key transcription factors was detected (Additional file 1: Fig. S4A, Additional file 4:Table S3), while subgroups I, II, and III were defined by a unique combination of master regulators (Additional file 1: Fig. S4A, Additional file 4:Table S3). To validate the specific TF activity per subgroup, we performed single-cell assay for transposase-accessible chromatin sequencing (scATAC-seq) on PHH incubated only with DMSO (Additional file 1: Fig. S4B). After scATAC-seq in one donor, we found that the promoter regions of subgroup-defining marker genes were open and accessible to the TFs predicted by ChEA3 in DMSO-treated cell (Additional file 1: Fig. S4, Methods).

### Primary human hepatocytes retain functional specialization in vitro in the absence of liver zonation

Among the four subgroups of hepatocytes identified in vitro, only subgroups I, II, and III were considered functional and metabolically active. We then further investigated whether these three hepatocyte subgroups could also be identified in vivo, using two distinct publicly available datasets [1, 5].

First, we investigated nine human livers described in Aizarani et al. in 2019 [1]. Hepatocytes were extracted from the data set based on the expression of mature hepatocyte maker genes *ALB*, *TTR*, and *HNF4A*. After performing *Louvain* clustering on the in vivo hepatocytes (resolution 0.2), we used the transcriptional profiles of our subgroups (I, II, and III) to identify hepatocyte specialization per cluster. We observed that the transcriptional profiles defining our subgroups are similarly expressed in hepatocytes in vivo with a correlation up to 0.94 for the top ten differentially expressed genes (DEGs) per subgroup (Fig. 1F and Additional file 1: Fig. S5).

In vivo, expression profiles of hepatocytes are affected by their position in the liver lobule [7, 57, 58]. Therefore, we investigated whether the three hepatocyte subgroups identified as subgroups I, II, and III in vivo were related to liver zonation patterns (Additional file 1: Fig. S5A). Based on their expression profile, cells were scored for zonation marker genes (Additional file 1: Fig. S5B, Methods) and assigned to three zones: pericentral, mid-zone, and periportal (Additional file 1: Fig. S5A and 5B). In subgroup I, 64.6% of cells were assigned to the periportal area, 6.6% to the midzone, and 46.5% to the pericentral area. The majority of cells in subgroup II, 52.3%, were assigned to the midzonal area, with 12.1% of cells in the pericentral area and 27.9% in the periportal area. Subgroup III showed an enrichment in mid- and pericentral expression profiles, 41.1% and 41.4%, respectively, and 7.5% in the periportal area (Additional file 1: Fig. S5B and 5C, Methods).

Additionally, hepatocyte subgroups and zonation were found to be intertwined in vivo (Additional file 1: Fig. S5D, left). For instance, *CYP27A1* in subgroup I was pericentrally enriched [59–61], while HS*D11B1*, involved in bile synthesis [62], was distributed periportally (Additional file 2: Table S1; Additional file 1: Fig. S5, left). Remarkably, in vitro, PHH retained specialization into the three functional subgroups (I, II, and III) in the absence of zonation (Additional file 2: Table S1; Additional file 1: Fig. S5, right). In subgroup I, the CY*P27A1* and *HSD11B1* had similar expression levels in all the cells. Similarly, zonation marker genes in subgroups II and III were evenly expressed in all cells studied.

These findings were extended to additional publicly available data sets for five human donors [5] (Additional file 1: Fig. S5E and 5F, Methods). In these five donors, MacParland et al*.* annotated six hepatocyte clusters, named Hep 1 to Hep 6 [5]. Based on marker gene overlaps, we found similarities in the gene expression profiles between those clusters and our hepatocyte subgroup in vitro.

In order to increase the power of the in vivo and in vitro comparisons, we computationally integrated the two in vivo datasets, resulting in a human cohort of 14 donors (Additional file 1: Fig. S5F). The identified metabolic subgroups showed high gene expression correlation between in vivo and in vitro (Additional file 1: Fig. S5G). In summary, our results using publicly available data sets from human donors indicate that PHH in vitro retained functional specialization in the absence of liver zonation.

### Phenotyping cocktail used to assess the induction of cytochrome P450 shows differential metabolic profiles among hepatocyte subgroups

In order to study the impact of cellular heterogeneity on liver function, we further characterized the hepatocyte subgroups by assessing their response to a phenotyping cocktail. The Sanofi-Aventis five-drug cocktail was used to simultaneously monitor the expression levels of the main five cytochrome P450 genes as a readout of the metabolic capacity of primary human hepatocytes [25, 63]. Therefore, this cocktail, consisting of a mixture of individual selective substrates of CYP2D6 (metoprolol), CYP2C19 (omeprazole), CYP2C9 (S-warfarin), CYP3A (midazolam), and CYP1A2 (caffeine), was used to monitor CYP induction [64] and incubated for 66 h in primary human hepatocytes (Methods).

**Fig. 2:**
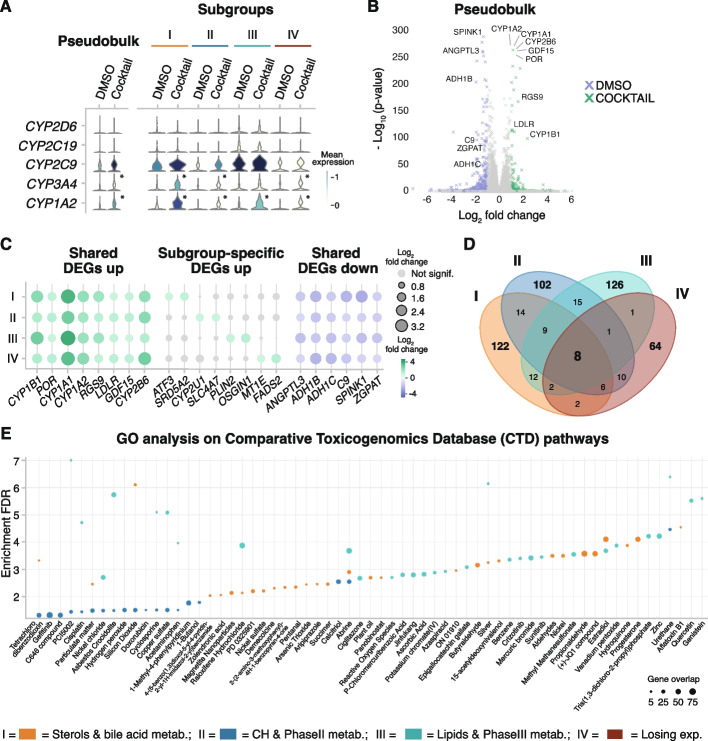
Subgroups of PHHs show different metabolic profile in response to 5-drug cocktail. **A** Violin plots depicting expression levels between Cocktail and DMSO of the five CYPs involved in the metabolism of the five-drug cocktail in pseudobulk (left), and in each hepatocyte subgroup (right) (* = *p*-value < 0.05 and |log_2_-fold change|> 1, *t*-test). **B** Volcano plot depicting the differential expression between Cocktail (green) and DMSO (purple) in pseudobulk. Text highlights the genes identified as DEGs in all subgroups. **C** Dot plot showing log_2_-fold change (color scale) and *p*-value (dot size) between Cocktail and DMSO for genes significantly up-regulated in all subgroups (left); representative subgroup-specific up-regulated genes (middle); and genes significantly down-regulated in all subgroups (right). **D** Venn diagram showing overlaps of significantly up-regulated genes upon cocktail treatment between the subgroups. **E** Scatter plot depicting enrichment of the genes specifically up-regulated in the metabolically active hepatocyte subgroups I, II, and III in pathways known to be involved in the metabolism of given chemical compounds (Drug.CTD database). The size of the dot corresponds to the number of overlapping genes in a given pathway

Incubation with the five-drug cocktail did not lead to substantial differences in the number of captured cells in each condition per subgroup (Additional file 1: Fig. S6A). Upon incubation, the induction of the mRNA of the five cytochrome P450 genes (CYPs) involved in the metabolism of the five-drug cocktail was monitored showing their up-regulation in pseudobulk (Fig. 2A left, Additional file 1: Fig. S6B). However, differences in the basal transcriptional levels were observed between subgroups showing transcriptional heterogeneity in a seemingly homogenous population of PHH (Fig. 2A, right). While the pseudobulk shows the expected mRNA basal levels of *CYP2C9* compared to *CYP1A2* and *CYP2D6* [65], scRNA-seq identified subgroups of hepatocytes with different basal mRNA levels of CYPs. Additionally, a subgroup-specific upregulation of CYPs upon cocktail incubation was also detected. For instance, in pseudobulk, *CYP2C9* was up-regulated 1.7-fold in Cocktail, while in subgroup III only a 1.1-fold change was detected. Likewise, *CYP3A4* was not significantly up-regulated in subgroup III, but a 4-fold increase was detected in subgroup I (Fig. 2A, right).

Thus, we wondered whether the subgroup of hepatocytes shared global transcriptional signatures in response to xenobiotics. We first analyzed global changes upon five-drug cocktail incubation, detecting 161 significantly up-regulated genes compared to DMSO (Fig. 2B and Additional file 1: Fig. S6C). All genes with a corrected *p*-value below 0.05 and a log_2_ fold change greater than 1 were designated as significantly up-regulated in cocktail-treated cells. Genes with log_2_ fold change below − 1 were considered significantly up-regulated in DMSO-treated cells (Fig. 2B and C, Additional file 1: Fig. S6D, Additional file 6: Table S5, Methods). Additionally, removing hepatocyte subgroup IV (characterized by the loss of expression) leads to the detection of 205 significantly up-regulated genes (Additional file 1: Fig. S6C, Additional file 5: Table S4).

Investigating the shared and subgroup-specific signatures in response to five-drug cocktail, only eight genes were significantly up-regulated upon cocktail treatment in all four subgroups: *CYP1B1*, *POR*, *CYP1A1*, *CYP1A2*, *RGS9*, *GDF15*, *CYP2A7*, and *CYP2B6* (Fig. 2B and C)*.* These genes were also detected as significantly up-regulated in the pseudobulk analysis and correspond to genes involved in the phase I metabolism of xenobiotics [20, 21].

We focused our attention on subgroup-specific DEGs that might be concealed by studies performed in bulk (Fig. 2C middle). We found subgroup-specific upregulated genes that were not detected as significantly up-regulated in the pseudobulk analysis. For example, upon five-drug cocktail incubation, hepatocytes in subgroup I (bile acid and sterol metabolism) specifically up-regulated *ATF3* and *SRD5A2*; subgroup II (carbohydrate and phase II metabolism) specifically up-regulated *CYP2U1* and *SLC4A7*; and subgroup III (lipids and phase III metabolism) specifically up-regulated *PLIN2* and *OSGIN* (Fig. 2C middle). A similar number of genes were specifically up-regulated in every metabolically active hepatocyte subgroup: 122 genes in subgroup I, 102 genes in subgroup II, and 126 genes in subgroup III. Meanwhile, only 64 genes were specifically up-regulated in subgroup IV (Fig. 2D). These results revealed subgroup-specific transcriptional signatures in response to xenobiotics that might be undetected in bulk studies.

Additionally, gene ontology (GO) analysis of subgroup-specific DEGs was performed using the Comparative Toxicogenomics Database (CTD) to explore toxicological interactions. This database is particularly suited for drug-disease or drug-phenotype interactions [66]. GO analysis using CTD showed that each hepatocyte subgroup specialized in the metabolism of certain xenobiotics, based on their differential transcriptomic profile (Fig. 2E, Additional file 1: Fig. S6E). For instance, the metabolic pathway of abrine was up-regulated across all hepatocyte subgroups I, II, and III. However, some compounds were only enriched in two subgroups, like cisplatin, in subgroups I and II. Finally, the pathways for the metabolism of other compounds were only enriched in one subgroup, like of ciglitazone and aflatoxin B1, enriched in subgroups II and I, respectively.

In summary, these results indicate that upon five-drug cocktail treatment, hepatocyte subgroups showed differential transcriptional responses, characterized by shared metabolic pathways and subgroup-specific transcriptional profiles associated with different potentials for metabolizing endobiotic (endogenous) and xenobiotic (exogenous) chemical compounds.

### Intracellular lipid accumulation leads to differential transcriptional variability among hepatocyte subgroups

Recently, it has been shown that changes in cytochrome P450 activity correlate with altered lipid metabolism [67–69]. For example, hepatic steatosis affects the transcriptomic profile of parenchymal and non-parenchymal cells as well as the cellular composition in the liver [32, 70, 71]. Particularly in hepatocytes, the lipid metabolism is disrupted upon fat accumulation through an alteration of key enzymes in the lipid synthesis, storage, and clearance pathways [72]. Furthermore, an increase in the production of chemokines has been observed, associated to the inflammation occurring in NAFLD [73].

**Fig. 3:**
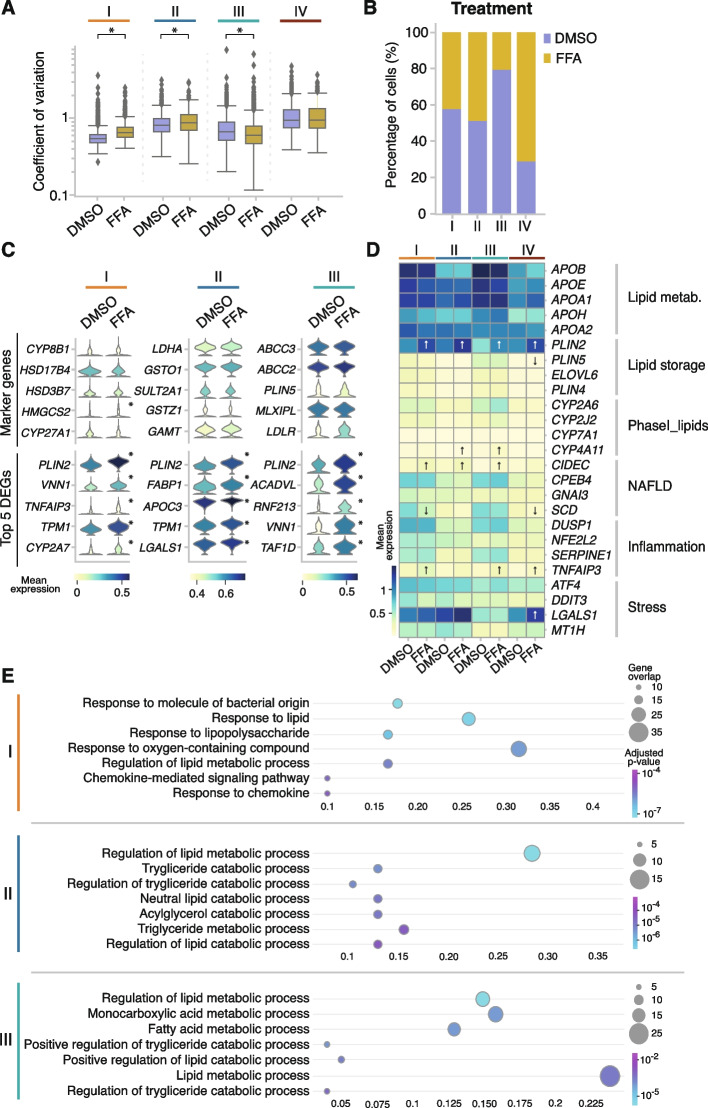
Intracellular lipid accumulation increases loss of expression and transcriptional variability. **A** Box plots depicting the coefficient of variation per gene in cells treated with DMSO or with FFA per subgroup (* = *p*-value < 0.05, Mann–Whitney *U*). **B** Bar plot showing the percentage of cells treated with DMSO or with FFA in every subgroup. **C** Stacked violin plots of the genes used in Fig. 1 to identify the subgroups, split into DMSO- and FFA-treated cells (top) and top 5 up-regulated genes upon fat accumulation per subgroup (bottom) in DMSO and FFA treatments (* = *p*-value < 0.05 and |log_2_-fold change|> 0.75, *t*-test). **D** Heatmap depicting the logarithmic mean expression of genes related to lipid metabolism, lipid storage, NAFLD-related genes, inflammation, and stress response in DMSO- and FFA-treated cells per subgroup (↑ indicates up-regulation towards DMSO; ↓ indicates down-regulation towards DMSO, *t*-test). **E** Scatter plot of the gene ontology (GO) analyses using *gprofiler* of the genes up-regulated upon fat accumulation in each of the subgroups. The top 7 most significantly enriched terms are depicted

To shed light on the effect of lipid accumulation on the metabolic capacity of functional subgroups of PHH, hepatic steatosis in vitro was modeled by incubating the cells with a 200-µM mixture of a 2:1 ratio of oleic acid to palmitic acid [35] (Methods). This mixture has previously been shown to mimic benign chronic steatosis with minor lipotoxic and apoptotic effects [35, 36]. First, we investigated whether — and to what extent — fat accumulation triggers a coordinated transcriptional response in PHHs. Thus, the coefficient of variation for DMSO- and FFA-treated cells was calculated per subgroup. Overall, cells losing expression (subgroup IV) showed the highest transcriptional variability (Fig. 3A, Additional file 1: Fig. S7A). Moreover, lipid accumulation significantly increased the variability in functional subgroups I and II, but decreased it in subgroup III (Fig. 3A). This indicated that subgroup III showed a more coordinated response towards accumulation of lipids.

Secondly, we observed that the proportion of cells treated with FFA differed between the functional hepatocyte subgroups. More than 74% of the cells in subgroup IV were FFA-treated cells, while in subgroup III 24% of the cells were FFA-treated cells. A similar percentage of FFA-treated cells were found in subgroups I and II (Fig. 3B, Additional file 1: Fig. S7B).

To explore global changes triggered by lipid accumulation, differential expression analysis was performed between FFA- and DMSO-treated cells. Compared to cocktail-treated cells, fewer up-regulated genes (3.5 times) were detected in FFA-treated cells over DMSO expression level (Additional file 1: Fig. S7C). Thus, genes with a corrected *p*-value below 0.05 and a log_2_ fold change greater than 0.75 were designated as significantly up-regulated in FFA-treated cells. Genes with log_2_ fold change below − 0.75 were considered significantly up-regulated in DMSO-treated cells (Additional file 1: Fig. S7C, Methods).

Thirdly, to track down the impact of intracellular fat accumulation on cellular identity, transcriptional changes in the previously selected marker genes — defining the hepatocyte subgroups — were investigated (Figs. 1F and 3C top). No significant changes in mean expression were observed for most of these marker genes upon accumulation of fat. Only *HMGCS2*, a key enzyme responsible for the synthesis of ketone bodies [74, 75] was significantly up-regulated in subgroup I upon fat accumulation (Fig. 3C, top).

In the metabolically active subgroups (I–III), the top five significantly up-regulated genes under fat accumulation were known markers of lipid droplet formation and lipid metabolism (Fig. 3C, bottom). For instance, the lipid droplet-associated perilipin protein *PLIN2* was significantly up-regulated in all subgroups, which has previously been shown to be relevant in diet-induced NAFLD [76, 77]. The inflammation marker *TNFAIP3*, also discovered to ameliorate NAFLD and be protective against its progression [78, 79], was up-regulated among the five top DEG genes in subgroup I (Fig. 3C).

Subsequent analysis of fat-metabolism-related pathways and genes involved in stress response [80] showed subgroup-specific signatures upon intracellular fat accumulation (Fig. 3D, and Additional file 3: Table S2 and Additional file 5: Table S4). For example, *CYP4A11*, involved in NAFLD progression by inducing ROS-related lipid peroxidation and inflammation [81, 82], was significantly up-regulated in subgroups II and III; and *CIDEC*, a promotor of triglyceride accumulation [83, 84], was significantly up-regulated in subgroups I, II, and III. Cells losing expression (IV), up-regulated *LGALS1* [85, 86], and *HSPB1* [87, 88] (Fig. 3D and Additional file 1: Fig. S6D), which are known markers in the gene ontology pathway GO:0006950: “response to stress.”

To identify the main biological processes associated to each metabolically active subgroup upon fat accumulation, gene ontology (GO) analyses were performed using all the significantly up-regulated genes per subgroup. Subgroup I showed gene overlaps in pathways related to cellular response to lipids, together with lipopolysaccharide and chemokine metabolism [89] (Fig. 3E). For instance, chemokines *CXCL8*, *CXCL1*, *CXCL10*, and *CXCL11* were up-regulated in FFA condition (Additional file 8: Table S7), suggesting that lipid accumulation could lead to increased inflammation through chemokine signaling [90, 91]. Moreover, subgroup II exhibited a high overlap of genes involved in the regulation of triglyceride metabolic processes, as well as in the acylglycerol catabolic process, denoting that these cells were rather involved in the clearance of neutral lipids [92, 93]. Finally, subgroup III cells showed enrichment in lipid, monocarboxylic acid, and fatty acid-related metabolic processes and lower transcriptional variability, most likely due to their coordinated response to fat accumulation [94–96].

Taken together, in metabolically active hepatocyte subgroups I and II, intracellular lipid accumulation increased transcriptional variability, thus affecting the fine-tuned regulation of lipid metabolism. Importantly, in subgroup III, specialized in the metabolism of lipids, transcriptional variability was reduced upon fat accumulation, suggesting a robust and tight coordinated response to chronic accumulation of lipids.

### Intracellular lipid accumulation impairs drug metabolism phases I, II, and III, with concomitant up-regulation of stress-related pathways

**Fig. 4:**
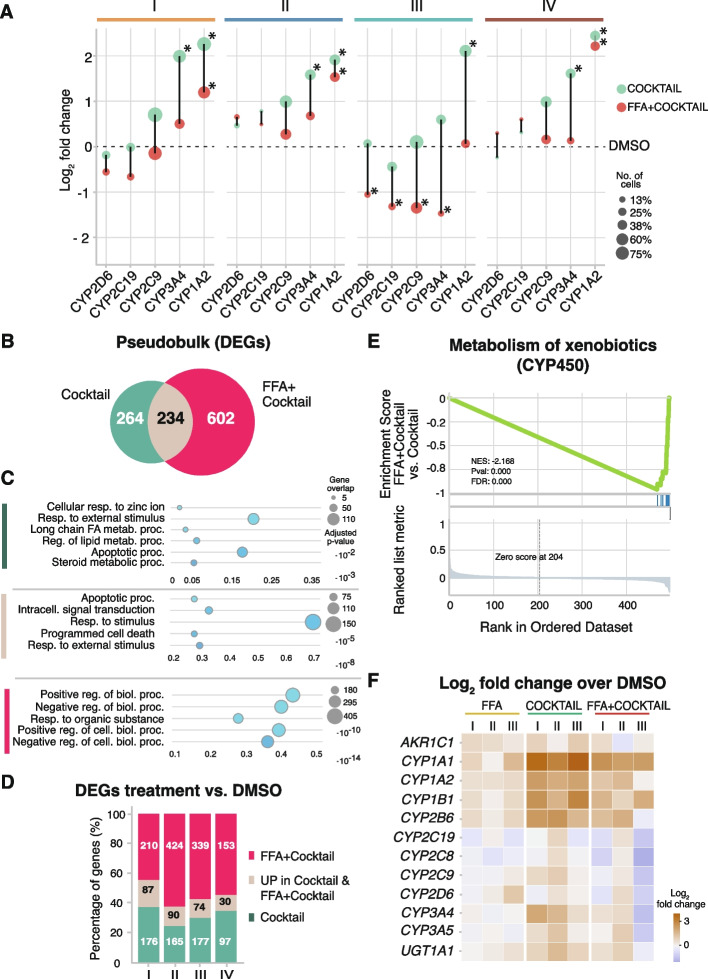
Intracellular lipid accumulation impairs drug metabolism phases I, II, and III. **A** Scatter plot depicting the log_2_-fold change of the 5 induced cytochromes between cocktail and DMSO (green), and between FFA + Cocktail and DMSO (red) in each subgroup (* = *p*-value < 0.05 and |log_2_-fold change|> 1, *t*-test). Dot size corresponds to the number of cells in which the gene is expressed. **B** Venn diagram showing the overlap of DEGs between Cocktail- vs. DMSO-treated cells (green) and FFA + Cocktail vs. DMSO-treated cells (red). **C** Scatter plot of the gene ontology (GO) analyses using *gprofiler* of the genes up-regulated specifically upon cocktail treatment (green); up-regulated specifically upon FFA + Cocktail treatment (magenta), and in both Cocktail and FFA + Cocktail (beige). The top 5 most significantly enriched terms are depicted. **D** Bar plot showing for each subgroup the percentages of genes specific to (a) Cocktail vs. DMSO (green); (b) genes up-regulated in both, Cocktail vs. DMSO and FFA + Cocktail vs. DMSO (beige); (c) specific to FFA + Cocktail vs. DMSO (magenta). **E** GSEA plot for FFA + Cocktail vs. Cocktail on the pathway of “Metabolism of xenobiotics by CYP450,” enriched in the Cocktail vs. DMSO-specific genes. **F** Heatmap depicting log_2_-fold change to DMSO level of genes involved in drug-metabolism related pathways that were enriched in Cocktail vs. FFA + Cocktail treatment

Large inter-individual variability has been observed in the metabolism of CYP substrates in vivo. This variability might be caused by genetic, epigenetic, and environmental factors. For instance, the simultaneous administration of five or more drugs, known as polypharmacy, is highly common in the clinical practice [38]. The co-administration of drugs increases the risk for developing hepatotoxicity and adverse drug reactions (ADRs), such as drug-induced liver injury (DILI) [97, 98]. Additionally, a higher incidence of DILI has been reported in patients suffering from NAFLD [41–43]. Therefore, we assessed the impact of fat accumulation in the phase I metabolism of drugs, using the previously characterized phenotyping cocktail (Sanofi-Aventis) (Fig. 2). The expression of the individual CYPs targeted by the drug cocktail was analyzed, comparing Cocktail- and FFA + Cocktail-treated cells (Fig. 4A). In all subgroups, fat accumulation decreased the expression levels of the five targeted cytochromes (Fig. 4A). For instance, in subgroups I, II, and IV, *CYP3A4* was significantly up-regulated upon cocktail treatment, but its induction was attenuated upon chronic lipid exposure. In subgroup III, the largest magnitude change of CYP expression was observed. Notably, *CYP2D6*, *CYP2C19*, *CYP2C9*, and *CYP3A4* were significantly down-regulated in comparison to baseline DMSO levels upon FFA + Cocktail treatment.

Additionally, to identify global transcriptional changes between Cocktail and FFA + Cocktail, differential expression analysis was performed in pseudobulk (Methods). Using DMSO as a baseline expression, 264 genes were up-regulated uniquely under Cocktail treatment; 234 genes were commonly up-regulated in both Cocktail treatment and FFA + Cocktail; and 602 genes were up-regulated solely in FFA + Cocktail, suggesting that more biological processes are affected (Fig. 4B, Additional file 1: Fig. S6D and S8A, Additional file 6: Table S5 and Additional file 9: Table S8). To further investigate the affected pathways, gene ontology (GO) analyses were performed on these genes (Fig. 4C). Specifically, upon Cocktail treatment, we observed an evident enrichment in pathways responsible for the metabolism of xenobiotic compounds (Fig. 4C, top, green). Genes commonly up-regulated upon Cocktail and FFA + Cocktail were less specific for drug metabolism and showed an enrichment in pathways for general stimulus responses (Fig. 4C, middle, beige). Finally, the genes specifically up-regulated in FFA + Cocktail showed an enrichment in stress-related pathways (Fig. 4C, bottom, magenta). The percentage of genes in each category was similar in all four subgroups of hepatocytes, suggesting that drug metabolism was similarly affected by the accumulation of fat (Fig. 4D). In addition, a down-regulation of key hepatic marker genes was observed in FFA + Cocktail (Additional file 1: Fig. S8B). For instance, *CYP2A7* was up-regulated in subgroups I, III, and IV upon Cocktail treatment, but the induction was impaired in the presence of fat (Additional file 1: Fig. S8B).

With the aim of dissecting the impact of chronic accumulation of fat on drug metabolism, we compared FFA + Cocktail and Cocktail treated cells in a gene set enrichment analysis (GSEA) (Methods) [99]. Among the top significant enriched pathways, the metabolism of xenobiotics by the CYP450 family was down-regulated (Fig. 4E), while the insulin resistance pathway was up-regulated in the presence of fat (Additional file 1: Fig. S8C), indicating a dysregulation of multiple metabolic pathways. For example, while chronic exposure to lipids did not trigger a consistent up-regulation of the insulin resistance pathway, the combination of FFA + Cocktail led to the up-regulation of *NFKBIA*, *TRIB3*, *CREB3L3*, *IRS2*, *SLC2A1*, *SOCS3*, *PIK3CD*, *CREB5*, *RELA*, and *CPT1B* in all subgroups (Additional file 1: Fig. S8D). Moreover, upon incubation with Cocktail, drug metabolism-related genes were up-regulated consistently in all functional subgroups of hepatocytes (I, II, II) (Fig. 4F). However, in the FFA + Cocktail treatment, subgroup III showed a drastic down-regulation in multiple phase I CYPs, e.g., *CYP2C19*, *CYP2C8*, *CYP2C9*, *CYP2D6*, *CYP3A4*, and *CYP3A5*; in phase II enzymes, *GSTA1*, *GSTA2*, and *SULT2A1*; and in phase III enzymes, *SLCO1B1* and *ABCG5*, exhibiting an overall impairment in all three phases of drug metabolism (Additional file 1: Fig. S9).

In summary, we observed that intracellular lipid accumulation led to an impairment of drug metabolism characteristics for each subgroup, in which multiple metabolic pathways are simultaneously affected. Therefore, primary human hepatocytes lose their drug-related metabolic specificity in the presence of chronic accumulation of lipids.

## Discussion

The application of single-cell genomics technologies allows the dissection of subtle differences within a seemingly homogeneous population of cells, which has been demonstrated in a plethora of organs, tissues, and cell types, including the liver [100–102]. The assessment of safety, toxicity, and efficacy of drugs is generally performed in bulk analyses, representing average features of the most abundant transcripts or the most abundant cell type [35, 50]. However, this approach hinders the assessment of cellular heterogeneity and the identification of cellular phenotypes that might be rare or display opposite transcriptional responses upon exposure to xenobiotics.

To circumvent this limitation, we have used single-cell transcriptomics to assess the metabolic profiles of individual PHHs, a gold standard human in vitro liver model, to study drug-related metabolic capacity in healthy condition and in response to environmental factors. Across all donors and treatment conditions, four major subgroups of hepatocytes were identified. Consistently with previous literature on PHHs [103–105], we identified a subgroup of PHHs losing the characteristic mature hepatocyte signature after 72 h in culture (subgroup IV, Fig. 1, Additional file 1: Fig. S2). For instance, in this subgroup, we observed a down-regulation of upstream liver-enriched transcription factors such as *MLXIPL* (*ChREBP*), *RXRA*, *NH1H4* (*FXR*), *PPARA*, *HNF4A*, and *CEBPA* [106, 107] (Additional file 1: Fig. S7B). Further analysis of metabolic profiles showed that subgroups I, II, and III were specialized in sterol and bile acid, carbohydrate and phase II, and lipids and phase III metabolism, respectively. Thus, we defined subgroups I, II, and III as metabolically active based on their respective transcriptional profiles (Fig. 1). Interestingly, in subgroup II, a high expression of stress markers was observed, suggesting that this group is prone to eventually lose mature hepatocyte-like expression (Additional file 1: Fig. S2) [108, 109]. This observation is supported by the high correlation between subgroup II and subgroup IV in vitro (Additional file 1: Fig. S4F). Moreover, the percentage of cells in each subgroup changed upon treatment, suggesting that fat accumulation and xenobiotics might change the number of cells per hepatocyte subgroup and the functional specialization of the liver tissue.

Another source of functional specialization is the well-known hepatic zonation in the liver. The zonation patterns found in vivo due to the oxygen and nutrient gradient along the lobule axis are not conserved in 2D in vitro models [91, 110–114]. To deeply investigate if hepatocyte subgroups are related to reminiscent liver zonation, we have compared our findings in vitro with two publicly available in vivo data sets comprising a cohort of 14 human donors [1, 5]. We have revealed the presence of the three metabolically active subgroups in vivo, mostly independent of zonation. Therefore, subgroup specialization and hepatocyte zonation might play both a key role in liver function. Since liver zonation has a profound impact in transcriptional regulation in individual cells [10], it is technically challenging to dissect the role of hepatocyte subgroups in vivo.

In vitro, we were able to assess the drug metabolic capacity of the hepatocyte subgroups by means of a well-known phenotyping cocktail (Fig. 2). The drug metabolism in vitro can be defined by three major phases. In phase I, oxidation, hydrolysis, reduction, and cyclization reactions are catabolized mainly by the cytochrome P450 (CYP450) superfamily of monooxygenase enzymes. The main members are the isoforms CYP1A2, 2C9, 2C19, 2D6, and 3A4, which account for the metabolism of around 70–80% of the clinically available drugs [19, 20]. This strategy, formally known as the “cocktail approach,” has been used to monitor the cytochrome enzymatic activity and changes in the induction of their corresponding mRNA levels in bulk [22–28]. We have selected the Sanofi-Aventis cocktail to dissect changes in the transcriptome of individual human hepatocytes. The Sanofi-Aventis cocktail can be used in different species, including mouse [63, 115], primates [116, 117], dogs and minipigs [116, 118], and humans, and in both in vivo [25, 63, 117] and in vitro studies [116].

With the Sanofi-Aventis cocktail, we have revealed transcriptomic responses in individual cells that were otherwise concealed in bulk studies. For instance, a high percentage of cells in subgroup IV might lead to an underestimated effect of drug induction, which is highly relevant in the safety evaluation of xenobiotics [119]. Additionally, exploration of toxicological interactions (i.e., CTD) showed that each hepatocyte subgroup is specialized in the metabolism of certain xenobiotics, which suggests that certain subgroups of hepatocytes could be more susceptible to develop adverse drug reactions (ADRs) and toxic metabolites when challenged by a specific compound (Fig. 2E). Furthermore, we anticipate that chronic liver diseases might also affect the percentage of cells in each hepatocyte subgroup and therefore have major implications in the assessment of drug efficacy, safety, and toxicity in the early phases of drug development.

To extend the impact of cellular heterogeneity in the human liver, we have mimicked hepatic steatosis and early stages of non-alcoholic fatty liver disease (NAFLD) by loading the cells with intracellular lipids (Fig. 3) [35, 120]. Chronic accumulation of fat led to an increase in transcriptional variability in subgroups I and II, indicating transcriptional noise and random fluctuation in the mRNA level in individual cells. Importantly, in subgroup III, specialized in the metabolism of lipids, transcriptional variability was reduced upon fat accumulation, suggesting a robust and tight coordinated response to chronic accumulation of lipids. In fact, it is well-known that after two-third partial hepatectomy, there is a prominent hepatic fat accumulation in the first round of DNA synthesis before mitotic activity [121, 122]. During partial hepatectomy-induced regeneration, four continuous waves of hepatic replication have been described as associated to a tight control on the number of hepatocytes entering into cell cycle. Interestingly, three waves of hepatic fat accumulation were coupled to hepatocyte replication [123], suggesting that lipid accumulation and cell cycle in hepatocytes are co-regulated processes. However, the precise molecular mechanism underlaying changes in cell cycle and fat accumulation still remains unknown [124].

It is also possible, that the lipid accumulation leads to increased inflammation in a specific cell population. Upon fat accumulation, we found subgroup-specific signatures in which lipopolysaccharide and chemokine metabolism were upregulated in subgroup I (Fig. 3). Subgroup I is the largest in our studied primary hepatocytes. These results reinforce the notion that changes in the proportion of hepatocyte subgroups might determine the functional outputs in response to environmental or dietary factors.

For instance, hepatic fat accumulation also occurs during heathy aging [125], and an age-related increase in transcriptional variability has been reported in several tissues and cell types [9, 94, 96, 126]. Additionally, aging affects the hepatocyte function [127] and cytochrome P450 enzymes [40, 128–130]. Therefore, further investigations on the age-associated cellular heterogeneity and drug metabolism could anticipate the unexpected adverse drug reactions or drug-induced liver toxicity in the elderly.

Additionally, in the elderly, the co-administration of drugs known as polypharmacy is highly common for the treatment of age-related comorbidities, increasing the risk for the development of ADRs and, more specifically, DILI [38, 39]. In all subgroups of hepatocytes, intracellular lipid accumulation diminished their capacity to metabolize the five-drug cocktail (Fig. 4). Especially, subgroup III, responsible for lipid and phase III metabolism, showed the most prominent decrease in the five targeted cytochromes simultaneously (Fig. 4).

## Conclusions

In summary, cellular heterogeneity is affected by intrinsic and extrinsic factors which might lead to aberrant metabolism, producing toxic metabolites and associated stress-related responses and inflammation. Intracellular lipid accumulation increases transcriptional variability and loss of expression in vitro, diminishing the capacity for drug metabolism in individual cells. Extrapolating these results to in vivo human biology, our findings would suggest that fat accumulation could further accelerate age-related processes by means of increased transcriptional noise and susceptibility to adverse drug reactions. Assessing the impact of cellular heterogeneity on tissue function will shed light on novel molecular mechanisms underlaying chronic or age-related pathologies.

## Methods

An outline of the experimental design is shown in Fig. 1. Briefly, commercially plateable and interaction-qualified cryopreserved human hepatocytes (Lonza, Walkersville, MD, USA) were purchased from Lonza from four different donors: HUM180812 (male, 57 years old, Hispanic) and HUM4152 (male, 18 years old, Caucasian), HUM181641 (male, 56 years old, Caucasian) and HUM4190 (male, 26 years old, Caucasian) (Additional file 2: Table S1). All donors had a body mass index in the normal range and were not diabetic (Additional file 2: Table S1). Cell lines were not authenticated and were not checked for mycoplasma contamination. The first two donors were processed simultaneously in a first batch, and the second two in a second batch, aiming for a maximum of eight samples processed at a time.

### Cell culture

Each cryovial of PHH was thawed and plated according to Lonza’s “Suspension and plateable cryopreserved hepatocytes: technical information and instructions.” the protocol was followed stepwise minutely, using the recommended thawing and plating media (Lonza, MCHT50, and MP250, respectively). The cells were dispensed and mixed using only wide orifice tips (Rainin, Ref. 17014297). For efficient cell seeding densities and attachment, cells were counted using the Trypan Blue Exclusion Method and seeded into Collagen-I coated plates at a density of approximately 100,000 cells/cm^2^ following Lonza’s instructions (Lonza, “Suspension and Plateable Cryopreserved Hepatocytes Technical Information and Instructions”). Six hours post-seeding, cells were washed with 1 mL of pre-warmed Maintenance Medium (Lonza, MCHT50) before the addition of treatment media. The treatment medium was renewed every 24 h for a total incubation period of 72 h post-seeding. Free fatty acids (FFA) consisting of a 200 µM mixture of a 2:1 ratio of the unsaturated oleic acid to the saturated palmitic acid were added to the maintenance media, to mimic the levels in human steatosis [35]. In order to facilitate FFA uptake, pre-bounding of free fatty acids to 1% bovine serum albumin in a 1:5 molar ratio (Sigma-Aldrich) was performed by heating the mixture at 40 °C for 2 h [36].

### Drug cocktail preparation and storage

The individual components of the 5-drug cocktail [24] were dissolved in sterile DMSO, filtered through a 0.2-µM syringe filter (Merck, SLGVV255F), and stored at − 80 °C for a maximum of six months (“compound stock concentration”, Additional file 11: Table S10). The individual drugs were mixed at 200 × concentration (“working concentration”, Additional file 11: Table S10) and added to the cells to a final concentration of 80 µM Caffeine (Sigma-Aldrich, Ref. 56396-100MG), 5 µM Midazolam (LGC Chemicals, Ref. LGCFOR1106.00), 17 µM Omeprazole (TRC Chemicals, Ref. 0635000), 20 µM S-Warfarin (Sigma-Aldrich, Ref. UC214-5MG) and 23 µM Metoprolol (TRC Chemicals, Ref. M338815). The final DMSO concentration used on the cells was 0.5% (v/v), in all conditions.

### Single-cell RNA-seq sample preparation and sequencing

After a total of 72-h incubation in a treatment culture medium, cells were detached with pre-warmed 0.25% Trypsin (Gibco, 25200056) for 7 min, followed by trypsin inactivation. The dissociated cells were then collected to pellet by centrifugation at 100 × *g* for 5 min at room temperature (RT). Cells were washed twice with 1 mL of pre-warmed 1 × PBS pH 7.4 (Gibco, 10010023), followed by cells pelleting at 100 × *g* for 5 min at RT. A live-cell selection was performed using a Dead Cell Removal Kit (Miltenyi Biotec, 130–090-101) as follows: cells were pelleted at 100 × *g* for 5 min at RT, resuspended in 100 µL of dead-cell removal microbeads and incubated for 15 min at RT. Dead cells were positively selected by passing the cell suspension through a MS column and performing a wash with a total of 2 mL of binding buffer. Living cells were eluted from the column and collected in 2-mL Eppendorf tubes. After pelleting by centrifugation at 100 × *g* for 5 min at RT, cells were resuspended in 1xPBS pH 7.4 supplemented with 0.04% BSA (Miltenyi Biotech, 130–091-376), stained with trypan blue to assess viability, and counted in a hemocytometer. A single-cell suspension was obtained by dissociating cells with wide orifice pipette tips and preparing the target cell stock concentration for loading the 10 × chip.

Single-cell RNA-seq libraries were prepared from each sample following the 10x Genomics Single Cell 3′ Reagent Kit User Guide (v3 or v3.1, manual CG000183 and CG000204, respectively) in the Chromium Controller (10x Genomics). The quality control of cDNA and obtained final libraries was performed using a Bioanalyzer High Sensitivity DNA Analysis assay (Agilent). Library quantification was performed using the Collibri™ Library Quantification Kit (Thermo Fischer Scientific, A38524500) in a QuantStudio™ 6 Flex Real-Time PCR System (Thermo Fisher Scientific). Both batches were sequenced in a NovaSeq6000 sequencer (Illumina) at the HMGU Core Facility for NGS Sequencing. The first batch (HUM180812 and HUM4152) was sequenced in a S2 flowcell at a depth of 250,000 reads per cell. The second batch (HUM181641 and HUM4190) was sequenced in a SP flowcell at a sequencing depth of 20,000 reads per cell. The sequencing length was set as indicated by 10x Genomics: 28/8/0/91.

### Single-cell ATAC-seq sample preparation and nuclei isolation

After a total of 72-h incubation in treatment culture medium (DMSO), cells were trypsinated with pre-warmed 0.25% Trypsin (Gibco, 25200056) for 7 min, followed by inactivation. Approximately 500,000 cells were pelleted at 100 × *g* for 5 min at RT. Cells were washed twice with 1 mL of cold 1 × PBS pH 7.4 (Gibco, 10010023), centrifuged down at 500 rcf for 5 min at 4 °C and resuspended in 1 mL of homogenization buffer (5 mM MgCl_2_, 25 mM KCl, 10 mM Tris buffer pH 8.0, 250 mM sucrose, 1% DTT, protease-inhibitor (Life Technologies, 1187358001), 0.2% NP-40 and 0.3% Triton-X). Cells were transferred to a 2-mL homogenizer and 5 strokes were performed with the lose pestle (A) followed by 10 min incubation on ice and 25 strokes with the tight pestle (B). Nuclei were then transferred to a 2-mL Eppendorf tube. The douncer was rinsed using additional 400 µL of HB and added to the tube, which was then centrifuged at 500 rcf for 5 min at 4 °C. After discarding the supernatant, the pellet was resuspended in 1 mL of swelling buffer (10 mM Tris–HCl pH7.5, 2 mM MgCl_2_, 3 mM CaCl_2_) and 1 mL of prechilled swelling buffer with 10% glycerol was added drop-wise and then pipette-mixed 5 times [131]. The mixture was incubated for 10 min on ice with occasional flicking. Then, the nuclei were pelleted and resuspended in 1 mL of 10 × Genomics Wash Buffer (10 mM Tris–HCl pH 7.4, 10 mM NaCl, MgCl_2_, 1% BSA, 0.1% Tween-20 and nuclease-free water), counted nuclei per µL and incubated for 15 min on ice. Proceeding to nuclei lysis, nuclei were centrifuged at 500 rcf at 4 °C for 5 min. Then, the supernatant discarded and nuclei resuspended in 200 µL of 10x Genomics Lysis Buffer with 0.2% NP-40 instead of the original 0.1% concentration (10 mM Tris–HCl pH 7.4, 10 mM NaCl, MgCl_2_, 1% BSA, 0.1%Tween-20, 0.01% Digitonin, as a minor 0.2% NP-40 and nuclease-free water). Nuclei were lyzed for 10 min on ice and then 1 mL of wash buffer was added followed by rocking the tube gently. Nuclei were pelleted at 500 rcf for 5 min at 4 °C, then 500 µL of PBS added without resuspending and nuclei spun down at 500 rcf for 5 min at 4 °C.

### Tagmentation, library preparation, and sequencing

Tagmentation reaction was prepared following the 10 × Genomics demonstrated protocol [131]. In brief, isolated nuclei were resuspended in 500 µL of 1X Nuclei Storage Buffer, pelleted at 500 rcf for 5 min at 4 °C, and discarding the supernatant. The transposition master mix was prepared by mixing 7 µL of ATAC Buffer and 3 µL of ATAC enzyme [131]. Then, using the Nuclei Concentration Guidelines calculator, 5 µL of the mixture of nuclei (2.9 µL) and diluted Nuclei Storage Buffer (2.1 µL) were incubated for 60 min at 37 °C in a thermocycler.

Single-cell ATAC-seq library was prepared following the 10x Genomics Chromium Next GEM Reagents Kit and Reagent Kit User Guide (v1.1, manual CG000209, RevD) [131] in the Chromium Controller (10x Genomics). The cDNA and library’s quality were assessed in a Bioanalyzer 2100 using the High Sensitivity DNA Analysis assay (Agilent). Final libraries’ quantification was performed using the Collibri™ Library Quantification Kit (Thermo Fischer Scientific, A38524500) in a QuantStudio™ 6 Flex Real-Time PCR System (Thermo Fisher Scientific). Sequencing was performed in a SP 100 flowcell in a NovaSeq 6000 sequencer (Illumina) at the HMGU Core Facility for NGS Sequencing with the sequencing length recommended by 10x Genomics (50, 8, 16, 50). Approximately, 50,000 reads per nucleus were yielded.

### Read alignment, counting, and filtering of the combined batches

Reads were aligned to GRCh38 and counted using 10x Genomics Cellranger 4.0.0 with standard parameters for each batch individually (Additional file 3: Table S2). The resulting count matrices were combined into a count matrix of 63,527 cells times 19,971 genes. Cells with at least 1000 counts and 500 genes were kept. Genes were kept if they were present in at least 5 cells and had fewer than 5 million reads. *Scrublet* [132] was used to identify doublets. Due to hepatocytes being subject to polyploidyzation, a lenient cutoff of 0.15 was used to avoid unwanted removal of tetraploid hepatocytes. This led to 1.7% of cells being annotated as doublets and subsequently being removed. Lastly, cells with more than 1% mitochondrial reads were removed, resulting in a filtered matrix of 49,378 cells times 16,256 genes.

### Normalization and initial clustering

*Scran* was used for library size normalization with parameter *min.mean* = *0.05*. After normalization, cells with more than 20,000 normalized counts were removed. *Scanpy* functions were used for calculating principal components (PCs) and clustering. Shortly, the top 50 PCs were used to construct a neighboring graph before calculating a UMAP embedding and *Louvain* clusters. For two samples, encapsulation of the cells in droplets partially failed during the 10X library preparation (wetting failure). Therefore, an initial *Louvain* resolution of 0.5 was used to verify that cells stemming from these samples clustered separately. Indeed, some cells from one of the failed samples clustered apart from the rest of the cells, and another two clusters showed fractal structures that were not present in the samples where encapsulation worked correctly. Hence, based on the knowledge of the wetting failure, these three clusters were removed, resulting in a final matrix of 38,232 cells times 16,256 genes. To remove batch effects in downstream analysis, the two batches were integrated using *Harmony* [48].

### Subgroup annotation based on individual donor analysis and clustering

In each of the four donors, the resolution of the *Louvain* clustering was selected so that every cluster contained every treatment condition (Additional file 1: Fig. S2A). Then, the top 1000 genes per *Louvain* cluster were calculated using *sc.tl.rank_genes_groups* with *n_genes* = *1000*. To find out which clusters were shared between donors, the percentage of overlaps between the top 1000 genes per cluster was calculated and hierarchical clustering was performed. By this approach, three distinct groups of similar *Louvain* clusters were detected between donors (Additional file 1: Fig. S1C). Two of the donor-specific clusters did not group together with clusters from other donors. Therefore, they were labeled as individual clusters to inspect where they would fall on the integrated data set (Additional file 1: Fig. S1C). Due to differences in sequencing depth, one group was identified in the first batch which was assigned to the cells losing expression. After integration and putting the group labels from the individual donor analysis on top of the combined UMAP, *Louvain* clustering was performed, showing that cells from the second batch that we had assigned to subgroup II were clustering with the cells losing their expression from the first batch. Therefore, those cells we re-labeled as losing their expression, subgroup IV (Additional file 1: Fig. S2A). One of the two donor-specific clusters that could initially not be assigned to a shared cluster was split into two clusters on the combined data set, which were assigned to subgroup I and subgroup II, respectively. The other individual cluster could be associated to the cells losing their expression. Marker genes were taken from reported literature to assign metabolic preferences to the functional subgroups I, II, and III (Additional file 3: Table S2).

### Comparison to publicly available in vivo data

Data were obtained from GEO (Accession number GSE124395) [1]. Genes with zero expression across all cells, and cells that did not express any genes, were removed. During further filtering, cells with 100 to 6000 genes and 800 to 30,000 reads were kept and genes were kept if they were covered in at least 10 cells, resulting in a count matrix of 11,059 cells times 19,416 genes. To keep the processing comparable to our data, normalization was performed using *scran* with parameter *min.mean* = *0.05*. After normalization, cells with more than 20,000 normalized counts were further removed, resulting in a count matrix of 11,043 cells times 19,416 genes. Clustering was performed using *scanpy* functions as described above. *Louvain* clustering was performed at a resolution of 0.08 to computationally separate the cell types in this data set from each other. Expression of mature hepatocyte markers, such as *ALB*, *HNF4A*, and *TTR* was used to identify the hepatocyte cluster. *Louvain* clustering with a resolution of 0.2 was then performed on the hepatocytes to identify hepatocyte subgroups in vivo. The expression of marker genes used for subgroup identification in our data was investigated on the *Louvain* clusters. Based on this marker gene expression, *Louvain* clusters were assigned to subgroups I, II, and III (Fig. 1D, Additional file 1: Fig. S5A).

As zonation impacts gene expression along the pericentral-periportal axis in vivo, we investigated its connection to our subgroups. Zonation markers were taken from Aizarani et al*.* (2019) [1]. Based on the 35 zones reported in their study, the genes were grouped into three zones, pericentral, mid, and periportal through binning. Then *sc.tl.score_genes* was used to calculate scores for each of the three zones (Additional file 1: Fig. S5C). Cells were assigned to pericentral [133] if they had a central vein (CV) score > 0.45 and a periportal (PV) score < 1. If cells had a PV score >  = 0.65 and a CV score <  = 0.45, they were assigned to periportal. The rest of the cells were assigned to mid-zone (Additional file 1: Fig. S5B and C). For each of the three subgroups, the percentage of cells in CV, mid, and PV was calculated subsequently (Additional file 1: Fig. S5B). For visualization purposes, the subgroups were separated and zonation marker genes for each subgroup were depicted on a UMAP for the in vivo data set and for our in vitro data (Additional file 1: Fig. S5D).

To further investigate the presence of our four hepatocyte subgroups in vivo, an additional human data set was downloaded from GEO (Accession number GSE115469) [5] filtered and normalized as before. In order to reduce potential noise, genes were removed if they were not present in at least three cells and counts were log-transformed for better comparability between datasets. Hepatocytes were isolated based on the authors’ annotation available at GSE115469. S*canpy* functions were used as described above to perform clustering with a *Louvain* resolution of 0.5 to separate subgroups of hepatocytes, leading to six *Louvain* clusters highly overlapping with the clusters reported in the initial study. Marker gene expression of the three identified subgroups in our data was again used to assign the six *Louvain* clusters to the three subgroups. To increase power, the two in vivo data sets were integrated using *scGen* [134] (Additional file 1: Fig. S4E). The top 10 DEGs per our in vitro subgroups were calculated and their scaled mean expression was correlated to the combined in vivo scaled mean expression. This showed a high correlation of marker genes between identified subgroups in vitro and in vivo (Additional file 1: Fig. S4F).

### Analysis of upstream regulators using ChEA3 and single-cell ATAC-seq

After defining metabolically functional subgroups in our in vitro data, we first calculated the top 500 DEGs per subgroup under DMSO in comparison to each other. To assess what drives the basal functional specialization of the subgroups, these top 500 genes per subgroup were taken as input for the online tool ChEA3 [53] which predict transcription factors regulating the gene expression per subgroup. For the purpose of visualization, five transcription factors among the top 25 per subgroup were depicted as a stacked violin plot (Additional file 1: Fig. S2E).

Raw reads were aligned to GRCh38 using *cellranger-atac 2.1.0*. Transcription factor binding site (TFBS) annotation was downloaded from ReMap2022 (https://remap2022.univ-amu.fr/) and filtered to only retain the binding sites of the top 25 transcription factors per subgroup that were predicted by ChEA3 (Additional file 4: Table S3). Using the fragment file obtained as output from *cellranger-arc*, we constructed the count matrix for these binding sites using epiScanpy’s function *epi.ct.peaks_mtx()*. This resulted in a matrix of 360,878 barcodes × 4,029,591 TFBS. Cells were removed if they had fewer than 2000 TFBS covered, a nucleosome signal score of > 5, and a TSS-enrichment score of < 2. TFBS were removed if they were present in fewer than 30 cells. Furthermore, highly variable TFBS were selected by running *epi.pp.highly_variable()* with parameter *min_score* = *0.515*. To only consider binding sites at close proximity of the subgroup-specific genes, TFBS were annotated by the genes in their proximity using *epi.tl.find_genes()* with parameters *upstream* = *1000* and *downstream* = *100*. We used this annotation to select only the TFBS falling within 1000 bp upstream and 100 bp downstream of the TSS of any of the top 500 DEGs per subgroup. This resulted in a final matrix of 3582 cells × 16,488 TFBS.

To confirm the presence of characteristic chromatin landscapes for our identified subgroups, we investigated the co-accessibility of the TFBS in the promoter regions of 5 genes per subgroup (subgroup I: *IQGAP2*, *LARP1*, *CTSB*, *DIAPH1*, *SPTBN1*; subgroup II: *RAC1*, *UGP2*, *RPL5*, *RPS12*, *ZNF706*; subgroup III: *OGT*, *ALB*, *CCNL1*, *NRBP2*, *PPP1R12B*). Briefly, the accessibility of each binding site across all cells was correlated to all others. Then, per TFBS, we calculated its average correlation to all the binding sites in the promoter of every selected gene. Clustering the TFBS based on these average correlations showed that TFBS at the promoters of subgroup-specific genes were clustering together. This indicates their co-accessibility in the cells (Additional file 1: Fig. S5A).

Additionally, *Louvain* clustering was applied using the above count matrix, to group cells showing similar openness profiles at the TFBS regions in front of subgroup-relevant genes. Cells that could not be assigned to one of the subgroups were disregarded. Finally, for the 1176 annotated cells, we visualized chromatin openness per subgroup at the binding sites of the ChEA3-predicted transcription factors in proximity to subgroup-defining genes (Additional file 1: Fig. S5B-D) [http://andrewjohnhill.com/blog/2019/04/12/streamlining-scatac-seq-visualization-and-analysis/)].

### Differential expression analysis between conditions in the subgroups

After defining metabolically functional subgroups in our in vitro data, we first calculated the top 500 DEGs per subgroup under DMSO in comparison to each other. To assess what drives the basal functional specialization of the subgroups, these top 500 genes per subgroup were taken as input for the online tool ChEA3 [53] which predict transcription factors regulating the gene expression per subgroup. For the purpose of visualization, five transcription factors among the top 25 per subgroup were depicted as a stacked violin plot (Additional file 1: Fig. S2E). To explore the implications of the functional specialization on the metabolic capacity of hepatocytes, we then performed differential expression analysis between Cocktail- and DMSO-treated cells in each of the functional subgroups. Genes were defined significantly up-regulated if they had a log_2_-fold change of greater than 1 and a Bonferroni-adjusted *p*-value below 0.05. We used the venn package to visualize the overlap of significantly up-regulated genes between the subgroups. Furthermore, ShinyGO [135] was used to investigate the enrichment of the subgroup-specific up-regulated genes in pathways known to be relevant for drug metabolism (Additional file 5: Table S4).

When comparing FFA- with DMSO-treated cells, we observed that there were on average 3.5 times fewer genes with a positive log_2_-fold change than when we compared Cocktail and DMSO. Therefore, to capture the subtler effects of fat accumulation on the cells, a gene was identified as significantly up-regulated if it had a Bonferroni-adjusted *p*-value below 0.05 and a log_2_-fold change greater than 0.75. We used the same marker genes as described above (Additional file 3: Table S2) to count overlaps between groups of marker genes and significantly up-regulated genes in the subgroups. To investigate in which pathways related to biological processed the genes up-regulated upon FFA-treatment were enriched in every subgroup, gprofiler was used (https://pypi.org/project/gprofiler-official/).

### Assessing transcriptional variability through the coefficient of variation

It is generally assumed that lowly expressed genes have an inflated transcriptional variability. Therefore, genes with a mean normalized log-transformed expression smaller than 0.25 were removed, and the coefficient of variation per condition in each of the subgroups was calculated on 3434 genes. To obtain the coefficient of variation on normalized, log-transformed counts, we used the formula described in Canchola et al. [136].

$$\mathrm{CV}=\sqrt{{e}^{{\sigma }^{2}}}-1,$$

where σ^2^ is the variation of gene *j* in the group of interest.

In every subgroup, a Mann–Whitney *U* test was performed to check if the coefficient of variation differed significantly between treatment condition.

### Supplementary Information

**Additional file 1: Fig. S1.** Quality controls. **Fig. S2.** Clustering and cell annotation. **Fig. S3.** Comparison between cell cycle analysis using FACS and Cyclone. **Fig. S4.** ChEA3 and scATAC-seq analysis. **Fig. S5.** *In vitro* and *in vivo* comparisons of hepatocyte subgroups. **Fig. S6.** Transcriptional signatures in response to the phenotyping cocktail. **Fig. S7.** Transcriptional profiles upon intracellular lipid accumulation. **Fig. S8.** Transcriptional dysregulation of multiple metabolic pathways upon fat accumulation. **Fig. S9.** Transcriptomic changes on phase III transporter genes.

**Additional file 2: Table S1.** Metadata donors.

**Additional file 3: Table S2.** Marker genes.

**Additional file 4: Table S3.** ChEA3 results per subgroup.

**Additional file 5: Table S4.** Unique DEGs per subgroup upon Cocktail.

**Additional file 6: Table S5.** Differential expression Cocktail vs. DMSO.

**Additional file 7: Table S6.** Significance levels coefficient of variation

**Additional file 8: Table S7.** Differential expression FFA vs. DMSO.

**Additional file 9: Table S8.** Differential expression FFA+Cocktail vs. DMSO.

**Additional file 10: Table S9.** Genes up-regulated upon Cocktail with and without FFA.

**Additional file 11: Table S10.** Drug Cocktail preparation

**Additional file 12.** Review history.

## Data Availability

All raw single-cell RNA sequencing data is deposited in ArrayExpress under accession numbers E-MTAB-11530 [137] and scATAC-seq under S-BSST1024 [138]. Additional publicly available data sets used for subgroup identification in vivo were obtained from GEO (Accession numbers GSE124395 [1, 139] and GSE115469 [5, 140]). The jupyter notebooks containing the code to reproduce the analysis results are publicly available via Github [141] and via Zenodo [142] and licensed under the GNU General Public License v3.0. Python libraries used for the analysis include scanpy (v1.7.2), anndata (v0.7.6), matplotlib (v3.4.1), pandas (v1.2.4), numpy (v1.19.2), seaborn (v0.11.1), and scipy (v1.6.2).

## References

[1] Aizarani N, Saviano A, Sagar, Mailly L, Durand S, Herman JS, Pessaux P, Baumert TF, Grün D (2019). A human liver cell atlas reveals heterogeneity and epithelial progenitors. Nature.

[2] Liao J, Yu Z, Chen Y, Bao M, Zou C, Zhang H, Liu D, Li T, Zhang Q, Li J (2020). Single-cell RNA sequencing of human kidney. Scientific Data.

[3] Litviňuková M, Talavera-López C, Maatz H, Reichart D, Worth CL, Lindberg EL, Kanda M, Polanski K, Heinig M, Lee M (2020). Cells of the adult human heart. Nature.

[4] Halpern KB, Shenhav R, Matcovitch-Natan O, Tóth B, Lemze D, Golan M, Massasa EE, Baydatch S, Landen S, Moor AE (2017). Single-cell spatial reconstruction reveals global division of labour in the mammalian liver. Nature.

[5] MacParland SA, Liu JC, Ma X-Z, Innes BT, Bartczak AM, Gage BK, Manuel J, Khuu N, Echeverri J, Linares I (2018). Single cell RNA sequencing of human liver reveals distinct intrahepatic macrophage populations. Nat Commun.

[6] Reyfman PA, Walter JM, Joshi N, Anekalla KR, McQuattie-Pimentel AC, Chiu S, Fernandez R, Akbarpour M, Chen C-I, Ren Z (2019). Single-cell transcriptomic analysis of human lung provides insights into the pathobiology of pulmonary fibrosis. Am J Respir Crit Care Med.

[7] Ramachandran P, Dobie R, Wilson-Kanamori JR, Dora EF, Henderson BEP, Luu NT, Portman JR, Matchett KP, Brice M, Marwick JA (2019). Resolving the fibrotic niche of human liver cirrhosis at single-cell level. Nature.

[8] Muraro MJ, Dharmadhikari G, Grün D, Groen N, Dielen T, Jansen E, van Gurp L, Engelse MA, Carlotti F, de Koning EJ, van Oudenaarden A (2016). A single-cell transcriptome atlas of the human pancreas. Cell Syst.

[9] Angelidis I, Simon LM, Fernandez IE, Strunz M, Mayr CH, Greiffo FR, Tsitsiridis G, Ansari M, Graf E, Strom T-M (2019). An atlas of the aging lung mapped by single cell transcriptomics and deep tissue proteomics. Nat Commun.

[10] Ben-Moshe S, Shapira Y, Moor AE, Manco R, Veg T, Bahar Halpern K, Itzkovitz S (2019). Spatial sorting enables comprehensive characterization of liver zonation. Nat Metab.

[11] Xiong X, Kuang H, Ansari S, Liu T, Gong J, Wang S, Zhao X-Y, Ji Y, Li C, Guo L (2019). Landscape of intercellular crosstalk in healthy and NASH liver revealed by single-cell secretome gene analysis. Mol Cell.

[12] Nault R, Fader KA, Bhattacharya S, Zacharewski TR (2021). Single-nuclei RNA sequencing assessment of the hepatic effects of 2,3,7,8-tetrachlorodibenzo-p-dioxin. Cell Mol Gastroenterol Hepatol.

[13] Castell JV, Jover R, Martinez-Jimenez CP, Gomez-Lechon MJ (2006). Hepatocyte cell lines: their use, scope and limitations in drug metabolism studies. Expert Opin Drug Metab Toxicol.

[14] Serras AS, Rodrigues JS, Cipriano M, Rodrigues AV, Oliveira NG, Miranda JP (2021). A critical perspective on 3D liver models for drug metabolism and toxicology studies. Front Cell Dev Biol.

[15] Garnier D, Li R, Delbos F, Fourrier A, Collet C, Guguen-Guillouzo C, Chesné C, Nguyen TH (2018). Expansion of human primary hepatocytes in vitro through their amplification as liver progenitors in a 3D organoid system. Sci Rep.

[16] Martínez-Jiménez CP, Gómez-Lechón MJ, Castell JV, Jover R (2006). Underexpressed coactivators PGC1alpha and SRC1 impair hepatocyte nuclear factor 4 alpha function and promote dedifferentiation in human hepatoma cells. J Biol Chem.

[17] Martinez-Jimenez, Ramiro J, Donato MT, Jose VC, Gomez-Lechon MJ (2007). Transcriptional regulation and expression of CYP3A4 in hepatocytes. Curr Drug Metab.

[18] Rodríguez-Antona C, Donato MT, Boobis A, Edwards RJ, Watts PS, Castell JV, Gómez-Lechón MJ (2002). Cytochrome P450 expression in human hepatocytes and hepatoma cell lines: molecular mechanisms that determine lower expression in cultured cells. Xenobiotica.

[19] Spatzenegger M, Jaeger W (1995). Clinical importance of hepatic cytochrome P450 in drug metabolism. Drug Metab Rev.

[20] Zanger UM, Schwab M (2013). Cytochrome P450 enzymes in drug metabolism: regulation of gene expression, enzyme activities, and impact of genetic variation. Pharmacol Ther.

[21] Rodriguez-Antona C, Donato MT, Pareja E, Gomez-Lechon MJ, Castell JV (2001). Cytochrome P-450 mRNA expression in human liver and its relationship with enzyme activity. Arch Biochem Biophys.

[22] Berger B, Bachmann F, Duthaler U, Krähenbühl S, Haschke M (2018). Cytochrome P450 enzymes involved in metoprolol metabolism and use of metoprolol as a CYP2D6 phenotyping probe drug. Front Pharmacol.

[23] Fuhr U, Jetter A, Kirchheiner J (2007). Appropriate phenotyping procedures for drug metabolizing enzymes and transporters in humans and their simultaneous use in the “cocktail” approach. Clin Pharmacol Ther.

[24] Ryu JY, Song IS, Sunwoo YE, Shon JH, Liu KH, Cha IJ, Shin JG (2007). Development of the “Inje cocktail” for high-throughput evaluation of five human cytochrome P450 isoforms in vivo. Clin Pharmacol Ther.

[25] Turpault S, Brian W, Van Horn R, Santoni A, Poitiers F, Donazzolo Y, Boulenc X (2009). Pharmacokinetic assessment of a five-probe cocktail for CYPs 1A2, 2C9, 2C19, 2D6 and 3A. Br J Clin Pharmacol.

[26] Bosilkovska M, Samer CF, Deglon J, Rebsamen M, Staub C, Dayer P, Walder B, Desmeules JA, Daali Y (2014). Geneva cocktail for cytochrome p450 and P-glycoprotein activity assessment using dried blood spots. Clin Pharmacol Ther.

[27] Chainuvati S, Nafziger AN, Leeder JS, Gaedigk A, Kearns GL, Sellers E, Zhang Y, Kashuba AD, Rowland E, Bertino JS Jr. Combined phenotypic assessment of cytochrome p450 1A2, 2C9, 2C19, 2D6, and 3A, N-acetyltransferase-2, and xanthine oxidase activities with the “Cooperstown 5+1 cocktail.” Clin Pharmacol Ther. 2003;74:437–47. 10.1016/S0009-9236(03)00229-7 14586384

[28] Christensen M, Andersson K, Dalén P, Mirghani RA, Muirhead GJ, Nordmark A, Tybring G, Wahlberg A, Yaşar U, Bertilsson L (2003). The Karolinska cocktail for phenotyping of five human cytochrome P450 enzymes. Clin Pharmacol Ther.

[29] Jancova P, Anzenbacher P, Anzenbacherova E (2010). Phase II drug metabolizing enzymes. Biomedical papers.

[30] Omiecinski CJ, Vanden Heuvel JP, Perdew GH, Peters JM (2011). Xenobiotic metabolism, disposition, and regulation by receptors: from biochemical phenomenon to predictors of major toxicities. Toxicol Sci.

[31] Park SR, Cho C-S, Xi J, Kang HM, Lee JH (2020). Holistic characterization of single-hepatocyte transcriptome responses to high-fat diet. Am J Physiol Endocrinol Metab.

[32] Su Q, Kim SY, Adewale F, Zhou Y, Aldler C, Ni M, Wei Y, Burczynski ME, Atwal GS, Sleeman MW (2021). Single-cell RNA transcriptome landscape of hepatocytes and non-parenchymal cells in healthy and NAFLD mouse liver. iScience.

[33] Younossi Z, Anstee QM, Marietti M, Hardy T, Henry L, Eslam M, George J, Bugianesi E (2018). Global burden of NAFLD and NASH: trends, predictions, risk factors and prevention. Nat Rev Gastroenterol Hepatol.

[34] Dulai PS, Singh S, Patel J, Soni M, Prokop LJ, Younossi Z, Sebastiani G, Ekstedt M, Hagstrom H, Nasr P (2017). Increased risk of mortality by fibrosis stage in nonalcoholic fatty liver disease: systematic review and meta-analysis. Hepatology.

[35] Gómez-Lechón MJ, Donato MT, Martínez-Romero A, Jiménez N, Castell JV, O’Connor JE. A human hepatocellular in vitro model to investigate steatosis. Chem Biol Interact. 2007;165:106–16. 10.1016/j.cbi.2006.11.004 17188672

[36] Kozyra M, Johansson I, Nordling Å, Ullah S, Lauschke VM, Ingelman-Sundberg M (2018). Human hepatic 3D spheroids as a model for steatosis and insulin resistance. Sci Rep.

[37] Seebacher F, Zeigerer A, Kory N, Krahmer N (2020). Hepatic lipid droplet homeostasis and fatty liver disease. Semin Cell Dev Biol.

[38] Mehta RS, Kochar BD, Kennelty K, Ernst ME, Chan AT (2021). Emerging approaches to polypharmacy among older adults. Nature Aging.

[39] Lavan AH, Gallagher P (2016). Predicting risk of adverse drug reactions in older adults. Ther Adv Drug Saf.

[40] Davies EA, O’Mahony MS. Adverse drug reactions in special populations – the elderly. Br J Clin Pharmacol. 2015;80:796–807. 10.1111/bcp.12596 PMC4594722 25619317

[41] Aubert J, Begriche K, Knockaert L, Robin MA, Fromenty B (2011). Increased expression of cytochrome P450 2E1 in nonalcoholic fatty liver disease: mechanisms and pathophysiological role. Clin Res Hepatol Gastroenterol.

[42] Begriche K, Massart J, Robin MA, Borgne-Sanchez A, Fromenty B (2011). Drug-induced toxicity on mitochondria and lipid metabolism: mechanistic diversity and deleterious consequences for the liver. J Hepatol.

[43] Tarantino G, Conca P, Basile V, Gentile A, Capone D, Polichetti G, Leo E (2007). A prospective study of acute drug-induced liver injury in patients suffering from non-alcoholic fatty liver disease. Hepatol Res.

[44] Xanthopoulos KG, Prezioso VR, Chen WS, Sladek FM, Cortese R, Darnell JE (1991). The different tissue transcription patterns of genes for HNF-1, C/EBP, HNF-3, and HNF-4, protein factors that govern liver-specific transcription. Proc Natl Acad Sci U S A.

[45] Stanulović VS, Kyrmizi I, Kruithof-de Julio M, Hoogenkamp M, Vermeulen JL, Ruijter JM, Talianidis I, Hakvoort TB, Lamers WH (2007). Hepatic HNF4alpha deficiency induces periportal expression of glutamine synthetase and other pericentral enzymes. Hepatology.

[46] Gómez-Lechón MJ, Tolosa L, Conde I, Donato MT (2014). Competency of different cell models to predict human hepatotoxic drugs. Expert Opin Drug Metab Toxicol.

[47] Sahi J, Grepper S, Smith C (2010). Hepatocytes as a tool in drug metabolism, transport and safety evaluations in drug discovery. Curr Drug Discov Technol.

[48] Korsunsky I, Millard N, Fan J, Slowikowski K, Zhang F, Wei K, Baglaenko Y, Brenner M, Loh PR, Raychaudhuri S (2019). Fast, sensitive and accurate integration of single-cell data with Harmony. Nat Methods.

[49] Gómez-Lechón MJ, Castell JV, Donato MT (2010). The use of hepatocytes to investigate drug toxicity. Methods Mol Biol.

[50] Heslop JA, Rowe C, Walsh J, Sison-Young R, Jenkins R, Kamalian L, Kia R, Hay D, Jones RP, Malik HZ (2017). Mechanistic evaluation of primary human hepatocyte culture using global proteomic analysis reveals a selective dedifferentiation profile. Arch Toxicol.

[51] Pelkonen O, Hakkola J, Hukkanen J, Turpeinen M (2020). CYP-associated drug–drug interactions: a mission accomplished?. Arch Toxicol.

[52] Scialdone A, Natarajan KN, Saraiva LR, Proserpio V, Teichmann SA, Stegle O, Marioni JC, Buettner F (2015). Computational assignment of cell-cycle stage from single-cell transcriptome data. Methods.

[53] Keenan AB, Torre D, Lachmann A, Leong AK, Wojciechowicz ML, Utti V, Jagodnik KM, Kropiwnicki E, Wang Z, Ma’ayan A (2019). ChEA3: transcription factor enrichment analysis by orthogonal omics integration. Nucleic Acids Res.

[54] Kyrmizi I, Hatzis P, Katrakili N, Tronche F, Gonzalez FJ, Talianidis I (2006). Plasticity and expanding complexity of the hepatic transcription factor network during liver development. Genes Dev.

[55] Odom Duncan T, Zizlsperger N, Gordon DB, Bell George W, Rinaldi Nicola J, Murray Heather L, Volkert Tom L, Schreiber J, Rolfe PA, Gifford David K (2004). Control of pancreas and liver gene expression by HNF transcription factors. Science.

[56] Eissing L, Scherer T, Tödter K, Knippschild U, Greve JW, Buurman WA, Pinnschmidt HO, Rensen SS, Wolf AM, Bartelt A (2013). De novo lipogenesis in human fat and liver is linked to ChREBP-β and metabolic health. Nat Commun.

[57] Dobie R, Wilson-Kanamori JR, Henderson BEP, Smith JR, Matchett KP, Portman JR, Wallenborg K, Picelli S, Zagorska A, Pendem SV (2019). Single-cell transcriptomics uncovers zonation of function in the mesenchyme during liver fibrosis. Cell Rep.

[58] Richter ML, Deligiannis IK, Yin K, Danese A, Lleshi E, Coupland P, Vallejos CA, Matchett KP, Henderson NC, Colome-Tatche M, Martinez-Jimenez CP (2021). Single-nucleus RNA-seq2 reveals functional crosstalk between liver zonation and ploidy. Nat Commun.

[59] Ben-Moshe S, Itzkovitz S (2019). Spatial heterogeneity in the mammalian liver. Nat Rev Gastroenterol Hepatol.

[60] Chen W, Suruga K, Nishimura N, Gouda T, Lam VN, Yokogoshi H (2005). Comparative regulation of major enzymes in the bile acid biosynthesis pathway by cholesterol, cholate and taurine in mice and rats. Life Sci.

[61] Vögeli I, Jung HH, Dick B, Erickson SK, Escher R, Funder JW, Frey FY, Escher G (2013). Evidence for a role of sterol 27-hydroxylase in glucocorticoid metabolism in vivo. J Endocrinol.

[62] Beck KR, Inderbinen SG, Kanagaratnam S, Kratschmar DV, Jetten AM, Yamaguchi H, Odermatt A (2019). 11β-Hydroxysteroid dehydrogenases control access of 7β,27-dihydroxycholesterol to retinoid-related orphan receptor γ. J Lipid Res.

[63] de Vries EM, Lammers LA, Achterbergh R, Klümpen HJ, Mathot RAA, Boelen A, Romijn JA (2016). Fasting-induced changes in hepatic P450 mediated drug metabolism are largely independent of the constitutive androstane receptor CAR. PLoS One.

[64] Drug Development and Drug Interactions | Table of Substrates, Inhibitors and Inducers. https://www.fda.gov/drugs/drug-interactions-labeling/drug-development-and-drug-interactions-table-substrates-inhibitors-and-inducers.

[65] Liu J, Lu YF, Corton JC, Klaassen CD (2021). Expression of cytochrome P450 isozyme transcripts and activities in human livers. Xenobiotica.

[66] Davis AP, Grondin CJ, Johnson RJ, Sciaky D, Wiegers J, Wiegers TC, Mattingly CJ (2021). Comparative Toxicogenomics Database (CTD): update 2021. Nucleic Acids Res.

[67] Fisher CD, Lickteig AJ, Augustine LM, Ranger-Moore J, Jackson JP, Ferguson SS, Cherrington NJ (2009). Hepatic cytochrome P450 enzyme alterations in humans with progressive stages of nonalcoholic fatty liver disease. Drug Metab Dispos.

[68] Donato MT, Lahoz A, Jiménez N, Pérez G, Serralta A, Mir J, Castell JV, Gómez-Lechón MJ (2006). Potential impact of steatosis on cytochrome P450 enzymes of human hepatocytes isolated from fatty liver grafts. Drug Metab Dispos.

[69] Greco D, Kotronen A, Westerbacka J, Puig O, Arkkila P, Kiviluoto T, Laitinen S, Kolak M, Fisher RM, Hamsten A (2008). Gene expression in human NAFLD. Am J Physiol Gastrointest Liver Physiol.

[70] Xiong X, Kuang H, Liu T, Lin JD (2020). A single-cell perspective of the mammalian liver in health and disease. Hepatology.

[71] Ægidius HM, Veidal SS, Feigh M, Hallenborg P, Puglia M, Pers TH, Vrang N, Jelsing J, Kornum BR, Blagoev B, Rigbolt KTG (2020). Multi-omics characterization of a diet-induced obese model of non-alcoholic steatohepatitis. Sci Rep.

[72] Cohen JC, Horton JD, Hobbs HH (2011). Human fatty liver disease: old questions and new insights. Science.

[73] Pan X, Chiwanda Kaminga A, Liu A, Wen SW, Chen J, Luo J (2020). Chemokines in non-alcoholic fatty liver disease: a systematic review and network meta-analysis. Front Immunol.

[74] Hegardt FG (1999). Mitochondrial 3-hydroxy-3-methylglutaryl-CoA synthase: a control enzyme in ketogenesis. Biochem J.

[75] Li J, Viswanadha S, Loor JJ (2012). Hepatic metabolic, inflammatory, and stress-related gene expression in growing mice consuming a low dose of trans-10, cis-12-conjugated linoleic acid. J Lipids.

[76] Imai Y, Varela GM, Jackson MB, Graham MJ, Crooke RM, Ahima RS (2007). Reduction of hepatosteatosis and lipid levels by an adipose differentiation-related protein antisense oligonucleotide. Gastroenterology.

[77] Imai Y, Boyle S, Varela GM, Caron E, Yin X, Dhir R, Dhir R, Graham MJ, Ahima RS (2012). Effects of perilipin 2 antisense oligonucleotide treatment on hepatic lipid metabolism and gene expression. Physiol Genomics.

[78] Liu D, Zhang P, Zhou J, Liao R, Che Y, Gao M-M, Sun J, Cai J, Cheng X, Huang Y (2020). TNFAIP3 interacting protein 3 overexpression suppresses nonalcoholic steatohepatitis by blocking TAK1 activation. Cell Metab.

[79] Zhang P, Wang P-X, Zhao L-P, Zhang X, Ji Y-X, Zhang X-J, Fang C, Lu Y-X, Yang X, Gao M-M (2018). The deubiquitinating enzyme TNFAIP3 mediates inactivation of hepatic ASK1 and ameliorates nonalcoholic steatohepatitis. Nat Med.

[80] Breher-Esch S, Sahini N, Trincone A, Wallstab C, Borlak J (2018). Genomics of lipid-laden human hepatocyte cultures enables drug target screening for the treatment of non-alcoholic fatty liver disease. BMC Med Genomics.

[81] Gao H, Cao Y, Xia H, Zhu X, Jin Y (2020). CYP4A11 is involved in the development of nonalcoholic fatty liver disease via ROS-induced lipid peroxidation and inflammation. Int J Mol Med.

[82] Powell PK, Wolf I, Lasker JM (1996). Identification of CYP4A11 as the major lauric acid ω-hydroxylase in human liver microsomes. Arch Biochem Biophys.

[83] Langhi C, Baldán Á (2015). CIDEC/FSP27 is regulated by peroxisome proliferator-activated receptor alpha and plays a critical role in fasting- and diet-induced hepatosteatosis. Hepatology (Baltimore, MD).

[84] Matsusue K, Kusakabe T, Noguchi T, Takiguchi S, Suzuki T, Yamano S, Gonzalez FJ (2008). Hepatic steatosis in leptin-deficient mice is promoted by the PPARγ target gene Fsp27. Cell Metab.

[85] Baek J-H, Kim D-H, Lee J, Kim S-J, Chun K-H (2021). Galectin-1 accelerates high-fat diet-induced obesity by activation of peroxisome proliferator-activated receptor gamma (PPARγ) in mice. Cell Death Dis.

[86] Liu F-T, Rabinovich GA (2005). Galectins as modulators of tumour progression. Nat Rev Cancer.

[87] Matsumoto T, Urushido M, Ide H, Ishihara M, Hamada-Ode K, Shimamura Y, Ogata K, Inoue K, Taniguchi Y, Taguchi T (2015). Small heat shock protein beta-1 (HSPB1) is upregulated and regulates autophagy and apoptosis of renal tubular cells in acute kidney injury. PLoS One.

[88] Long S, Peng F, Song B, Wang L, Chen J, Shang B (2021). Heat shock protein beta 1 is a prognostic biomarker and correlated with immune infiltrates in hepatocellular carcinoma. Int J Gen Med.

[89] Bode JG, Albrecht U, Häussinger D, Heinrich PC, Schaper F (2012). Hepatic acute phase proteins – regulation by IL-6- and IL-1-type cytokines involving STAT3 and its crosstalk with NF-κB-dependent signaling. Eur J Cell Biol.

[90] Feng J, Wei T, Cui X, Wei R, Hong T (2021). Identification of key genes and pathways in mild and severe nonalcoholic fatty liver disease by integrative analysis. Chronic Dis Transl Med.

[91] Zhang X, Shen J, Man K, Chu ESH, Yau TO, Sung JCY, Go MYY, Deng J, Lu L, Wong VWS (2014). CXCL10 plays a key role as an inflammatory mediator and a non-invasive biomarker of non-alcoholic steatohepatitis. J Hepatol.

[92] Schulze RJ, Drižytė K, Casey CA, McNiven MA (2017). Hepatic lipophagy: new insights into autophagic catabolism of lipid droplets in the liver. Hepatol Commun.

[93] Barbosa AD, Siniossoglou S (2017). Function of lipid droplet-organelle interactions in lipid homeostasis. Biochim Biophys Acta Mol Cell Res.

[94] Martinez-Jimenez CP, Eling N, Chen HC, Vallejos CA, Kolodziejczyk AA, Connor F, Stojic L, Rayner TF, Stubbington MJT, Teichmann SA (2017). Aging increases cell-to-cell transcriptional variability upon immune stimulation. Science.

[95] Kolodziejczyk AA, Kim JK, Tsang JC, Ilicic T, Henriksson J, Natarajan KN, Tuck AC, Gao X, Bühler M, Liu P (2015). Single cell RNA-sequencing of pluripotent states unlocks modular transcriptional variation. Cell Stem Cell.

[96] Enge M, Arda HE, Mignardi M, Beausang J, Bottino R, Kim SK, Quake SR (2017). Single-cell analysis of human pancreas reveals transcriptional signatures of aging and somatic mutation patterns. Cell.

[97] Benesic A, Jalal K, Gerbes AL (2019). Drug-drug combinations can enhance toxicity as shown by monocyte-derived hepatocyte-like cells from patients with idiosyncratic drug-induced liver injury. Toxicol Sci.

[98] Juurlink DN, Mamdani M, Kopp A, Laupacis A, Redelmeier DA (2003). Drug-drug interactions among elderly patients hospitalized for drug toxicity. JAMA.

[99] Subramanian A, Tamayo P, Mootha VK, Mukherjee S, Ebert BL, Gillette MA, Paulovich A, Pomeroy SL, Golub TR, Lander ES, Mesirov JP (2005). Gene set enrichment analysis: a knowledge-based approach for interpreting genome-wide expression profiles. Proc Natl Acad Sci.

[100] Payen VL, Lavergne A, Alevra Sarika N, Colonval M, Karim L, Deckers M, Najimi M, Coppieters W, Charloteaux B, Sokal EM, El Taghdouini A (2021). Single-cell RNA sequencing of human liver reveals hepatic stellate cell heterogeneity. JHEP Rep.

[101] Massalha H, Bahar Halpern K, Abu-Gazala S, Jana T, Massasa EE, Moor AE, Buchauer L, Rozenberg M, Pikarsky E, Amit I (2020). A single cell atlas of the human liver tumor microenvironment. Mol Syst Biol.

[102] Andrews TS, Atif J, Liu JC, Perciani CT, Ma XZ, Thoeni C, et al. Single-cell, single-nucleus, and spatial RNA sequencing of the human liver identifies cholangiocyte and mesenchymal heterogeneity. Hepatol Commun. 2022;6(4):821–40. 10.1002/hep4.1854. 10.1002/hep4.1854 PMC8948611 34792289

[103] Vinci B, Duret C, Klieber S, Gerbal-Chaloin S, Sa-Cunha A, Laporte S, Suc B, Maurel P, Ahluwalia A, Daujat-Chavanieu M (2011). Modular bioreactor for primary human hepatocyte culture: medium flow stimulates expression and activity of detoxification genes. Biotechnol J.

[104] Godoy P, Hewitt NJ, Albrecht U, Andersen ME, Ansari N, Bhattacharya S, Bode JG, Bolleyn J, Borner C, Böttger J (2013). Recent advances in 2D and 3D in vitro systems using primary hepatocytes, alternative hepatocyte sources and non-parenchymal liver cells and their use in investigating mechanisms of hepatotoxicity, cell signaling and ADME. Arch Toxicol.

[105] Hewitt NJ, Lechón MJ, Houston JB, Hallifax D, Brown HS, Maurel P, Kenna JG, Gustavsson L, Lohmann C, Skonberg C (2007). Primary hepatocytes: current understanding of the regulation of metabolic enzymes and transporter proteins, and pharmaceutical practice for the use of hepatocytes in metabolism, enzyme induction, transporter, clearance, and hepatotoxicity studies. Drug Metab Rev.

[106] Odom DT, Dowell RD, Jacobsen ES, Gordon W, Danford TW, MacIsaac KD, Rolfe PA, Conboy CM, Gifford DK, Fraenkel E (2007). Tissue-specific transcriptional regulation has diverged significantly between human and mouse. Nat Genet.

[107] Hayhurst GP, Lee YH, Lambert G, Ward JM, Gonzalez FJ (2001). Hepatocyte nuclear factor 4alpha (nuclear receptor 2A1) is essential for maintenance of hepatic gene expression and lipid homeostasis. Mol Cell Biol.

[108] Aitken AE, Richardson TA, Morgan ET (2006). Regulation of drug-metabolizing enzymes and transporters in inflammation. Annu Rev Pharmacol Toxicol.

[109] Renton KW (2005). Regulation of drug metabolism and disposition during inflammation and infection. Expert Opin Drug Metab Toxicol.

[110] Scheidecker B, Shinohara M, Sugimoto M, Danoy M, Nishikawa M, Sakai Y (2020). Induction of in vitro metabolic zonation in primary hepatocytes requires both near-physiological oxygen concentration and flux. Front Bioeng Biotechnol.

[111] Tonon F, Giobbe GG, Zambon A, Luni C, Gagliano O, Floreani A, Grassi G, Elvassore N (2019). In vitro metabolic zonation through oxygen gradient on a chip. Sci Rep.

[112] Wahlicht T, Vièyres G, Bruns SA, Meumann N, Büning H, Hauser H, Schmitz I, Pietschmann T, Wirth D (2020). Controlled functional zonation of hepatocytes in vitro by engineering of wnt signaling. ACS Synth Biol.

[113] Kang YB, Eo J, Mert S, Yarmush ML, Usta OB (2018). Metabolic patterning on a chip: towards in vitro liver zonation of primary rat and human hepatocytes. Sci Rep.

[114] Danoy M, Poulain S, Lereau-Bernier M, Kato S, Scheidecker B, Kido T, Miyajima A, Sakai Y, Plessy C, Leclerc E (2020). Characterization of liver zonation-like transcriptomic patterns in HLCs derived from hiPSCs in a microfluidic biochip environment. Biotechnol Prog.

[115] Yamazaki Y, Moore R, Negishi M (2011). Nuclear receptor CAR (NR1I3) is essential for DDC-induced liver injury and oval cell proliferation in mouse liver. Lab Invest.

[116] Shida S, Yamazaki H (2016). Human plasma concentrations of five cytochrome P450 probes extrapolated from pharmacokinetics in dogs and minipigs using physiologically based pharmacokinetic modeling. Xenobiotica.

[117] Koyanagi T, Nakanishi Y, Murayama N, Yamaura Y, Ikeda K, Yano K, Uehara S, Utoh M, Kim S, Uno Y, Yamazaki H (2015). Age-related changes of hepatic clearances of cytochrome P450 probes, midazolam and R-/S-warfarin in combination with caffeine, omeprazole and metoprolol in cynomolgus monkeys using in vitro–in vivo correlation. Xenobiotica.

[118] Mogi M, Toda A, Iwasaki K, Kusumoto S, Takehara H, Shimizu M, Murayama N, Izumi H, Utoh M, Yamazaki H (2012). Simultaneous pharmacokinetics assessment of caffeine, warfarin, omeprazole, metoprolol, and midazolam intravenously or orally administered to Microminipigs. J Toxicol Sci.

[119] Hakkola J, Hukkanen J, Turpeinen M, Pelkonen O (2020). Inhibition and induction of CYP enzymes in humans: an update. Arch Toxicol.

[120] Tolosa L, Gómez-Lechón MJ, Jiménez N, Hervás D, Jover R, Donato MT (2016). Advantageous use of HepaRG cells for the screening and mechanistic study of drug-induced steatosis. Toxicol Appl Pharmacol.

[121] Liao Y, Shikapwashya ON, Shteyer E, Dieckgraefe BK, Hruz PW, Rudnick DA (2004). Delayed hepatocellular mitotic progression and impaired liver regeneration in early growth response-1-deficient mice*. J Biol Chem.

[122] Stein TA, Burns GP, Tropp BE, Wise L (1985). Hepatic fat accumulation during liver regeneration. J Surg Res.

[123] Zou Y, Bao Q, Kumar S, Hu M, Wang GY, Dai G (2012). Four waves of hepatocyte proliferation linked with three waves of hepatic fat accumulation during partial hepatectomy-induced liver regeneration. PLoS One.

[124] Caldez MJ, Bjorklund M, Kaldis P (2020). Cell cycle regulation in NAFLD: when imbalanced metabolism limits cell division. Hepatol Int.

[125] Ogrodnik M, Miwa S, Tchkonia T, Tiniakos D, Wilson CL, Lahat A, Day CP, Burt A, Palmer A, Anstee QM (2017). Cellular senescence drives age-dependent hepatic steatosis. Nat Commun.

[126] Bahar R, Hartmann CH, Rodriguez KA, Denny AD, Busuttil RA, Dolle ME, Calder RB, Chisholm GB, Pollock BH, Klein CA, Vijg J (2006). Increased cell-to-cell variation in gene expression in ageing mouse heart. Nature.

[127] Acun A, Oganesyan R, Uygun K, Yeh H, Yarmush ML, Uygun BE (2021). Liver donor age affects hepatocyte function through age-dependent changes in decellularized liver matrix. Biomaterials.

[128] Abdelmegeed MA, Choi Y, Ha SK, Song BJ (2016). Cytochrome P450–2E1 promotes aging-related hepatic steatosis, apoptosis and fibrosis through increased nitroxidative stress. Free Radic Biol Med.

[129] Li CY, Renaud HJ, Klaassen CD, Cui JY (2016). Age-specific regulation of drug-processing genes in mouse liver by ligands of xenobiotic-sensing transcription factors. Drug Metab Dispos.

[130] Song G, Sun X, Hines RN, McCarver DG, Lake BG, Osimitz TG, Creek MR, Clewell HJ, Yoon M (2017). Determination of human hepatic CYP2C8 and CYP1A2 age-dependent expression to support human health risk assessment for early ages. Drug Metab Dispos.

[131] Genomics X. Chromium single cell ATAC reagent kits user guide (v1.1 Chemistry). 2021.

[132] Wolock SL, Lopez R, Klein AM (2019). Scrublet: computational identification of cell doublets in single-cell transcriptomic data. Cell Syst.

[133] Thummel KE, Shen DD, Podoll TD, Kunze KL, Trager WF, Bacchi CE, Marsh CL, McVicar JP, Barr DM, Perkins JD (1994). Use of midazolam as a human cytochrome P450 3A probe: II. Characterization of inter- and intraindividual hepatic CYP3A variability after liver transplantation. J Pharmacol Exp Ther.

[134] Lotfollahi M, Wolf FA, Theis FJ (2019). scGen predicts single-cell perturbation responses. Nat Methods.

[135] Ge SX, Jung D, Yao R (2019). ShinyGO: a graphical gene-set enrichment tool for animals and plants. Bioinformatics.

[136] Canchola J (2017). Correct use of percent coefficient of variation (%CV) formula for log-transformed data. MedCrave Online J Proteomics Bioinform.

[137] Sanchez-Quant E, Richter ML, Colomé-Tatché M, Martinez-Jimenez CP. Single-cell metabolic profiling reveals subgroups of primary human hepatocytes with heterogeneous responses to drug challenge. BioStudies, E-MTAB-11530 2023, https://www.ebi.ac.uk/biostudies/arrayexpress/studies/E-MTAB-11530. 10.1186/s13059-023-03075-9 PMC10583437 37848949

[138] Sanchez-Quant E, Richter ML, Colomé-Tatché M, Martinez-Jimenez CP. Single-cell metabolic profiling reveals subgroups of primary human hepatocytes with heterogeneous responses to drug challenge. BioStudies, S-BSST1024. 2023, https://www.ebi.ac.uk/biostudies/studies/S-BSST1024. 10.1186/s13059-023-03075-9 PMC10583437 37848949

[139] Aizarani N, Saviano A, Sagar, Mailly L, Durand S, Herman JS, Pessaux P, Baumert TF, Grün D. A human liver cell atlas reveals heterogeneity and epithelial progenitors. Datasets Gene Expression Omnibus. 2019, https://identifiers.org/geo:GSE124395. 10.1038/s41586-019-1373-2 PMC6687507 31292543

[140] MacParland SA, Liu JC, Ma X-Z, Innes BT, Bartczak AM, Gage BK, Manuel J, Khuu N, Echeverri J, Linares I, et al. Single cell RNA sequencing of human liver reveals distinct intrahepatic macrophage populations. Datasets Gene Expression Omnibus. 2018, https://identifiers.org/geo:GSE115469. 10.1038/s41467-018-06318-7 PMC6197289 30348985

[141] Sanchez-Quant E, Richter ML, Colomé-Tatché M, Martinez-Jimenez CP. Single-cell metabolic profiling reveals subgroups of primary human hepatocytes with heterogeneous responses to drug challenge. Github. 2023, https://github.com/celiamtnez/precision_toxicology.git. 10.1186/s13059-023-03075-9 PMC10583437 37848949

[142] Sanchez-Quant E, Richter ML, Colomé-Tatché M, Martinez-Jimenez CP. Single-cell metabolic profiling reveals subgroups of primary human hepatocytes with heterogeneous responses to drug challenge. Zenodo. 2023, 10.5281/zenodo.8256355. 10.1186/s13059-023-03075-9 PMC10583437 37848949

