# Supplementary material for: Single-cell metabolic profiling reveals subgroups of primary human hepatocytes with heterogeneous responses to drug challenge
Source: Genome Biol. 2023 Oct 17;24:234. doi: 10.1186/s13059-023-03075-9 (PMC10583437; doi:10.1186/s13059-023-03075-9)
Supplement: Supplementary file 1 — Additional file 1: Fig. S1. Quality controls. Fig. S2. Clustering and cell annotation. Fig. S3. Comparison between cell cycle analysis using FACS and Cyclone. Fig. S4. ChEA3 and scATAC-seq analysis. Fig. S5. In vitro and in vivo comparisons of hepatocyte subgroups. Fig. S6. Transcriptional signatures in response to the phenotyping cocktail. Fig. S7. Transcriptional profiles upon intracellular lipid accumulation. Fig. S8. Transcriptional dysregulation of multiple metabolic pathways upon fat accumulation. Fig. S9. Transcriptomic changes on phase III transporter genes. [file 13059_2023_3075_MOESM1_ESM.pdf]

SUPPLEMENTARY INFORMATION

Single-cell metabolic profiling reveals subgroups of primary human hepatocytes with heterogeneous responses to drug challenge.

E. Sanchez-Quant[1*], M. L. Richter[1*], M. Colomé-Tatché[2,3,4,†], C.P. Martinez-Jimenez[1,5,†]

[1] Helmholtz Pioneer Campus (HPC), Helmholtz Zentrum München. 85764 Neuherberg, Germany.

[2] Institute of Computational Biology (ICB), Helmholtz Zentrum München. 85764 Neuherberg, Germany.

[3] TUM School of Life Sciences Weihenstephan, Technical University of Munich (TUM). 85354 Freising, Germany.

[4] Biomedical Center (BMC), Physiological Chemistry, Faculty of Medicine, Ludwig Maximilian University of Munich (LMU). 82152 Munich, Germany.

[5] TUM School of Medicine, Technical University of Munich. Munich (TUM). 80333 Munich, Germany.

[*] These authors contributed equally to this work
† Corresponding author

Figure S1

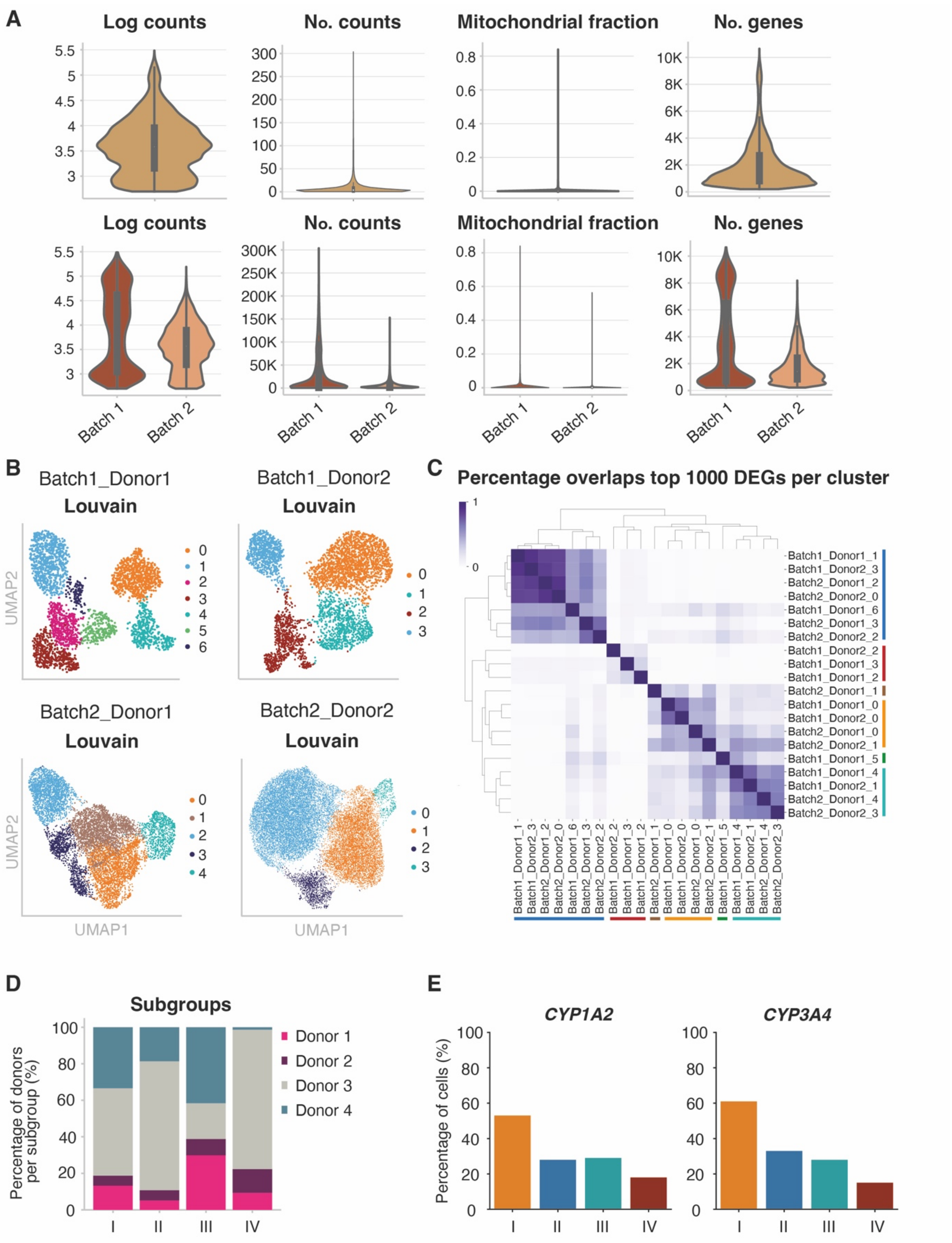

Figure S1: Quality controls. (A) Quality control violin plots on – from left to right – i) number of logarithmic counts per cell, ii) percentage of mitochondrial reads per cell, iii) number of genes per cell, iv) number of normalized counts per cell (top row: on all cells together, bottom row: separated by batch). **(B)** UMAPs showing *Louvain* clustering for each of the 4 donors. **(C)** Heatmap showing the percentage of overlaps of the top 1000 genes per cluster between the

Louvain clusters from all donors. **(D)** Barplot depicting the percentage of cells from each donor in the four hepatocyte subgroups. **(E)** Barplot showing the percentage of cells expression CYP1A2 and 3A4 per hepatocyte subgroup respectively

Figure S2

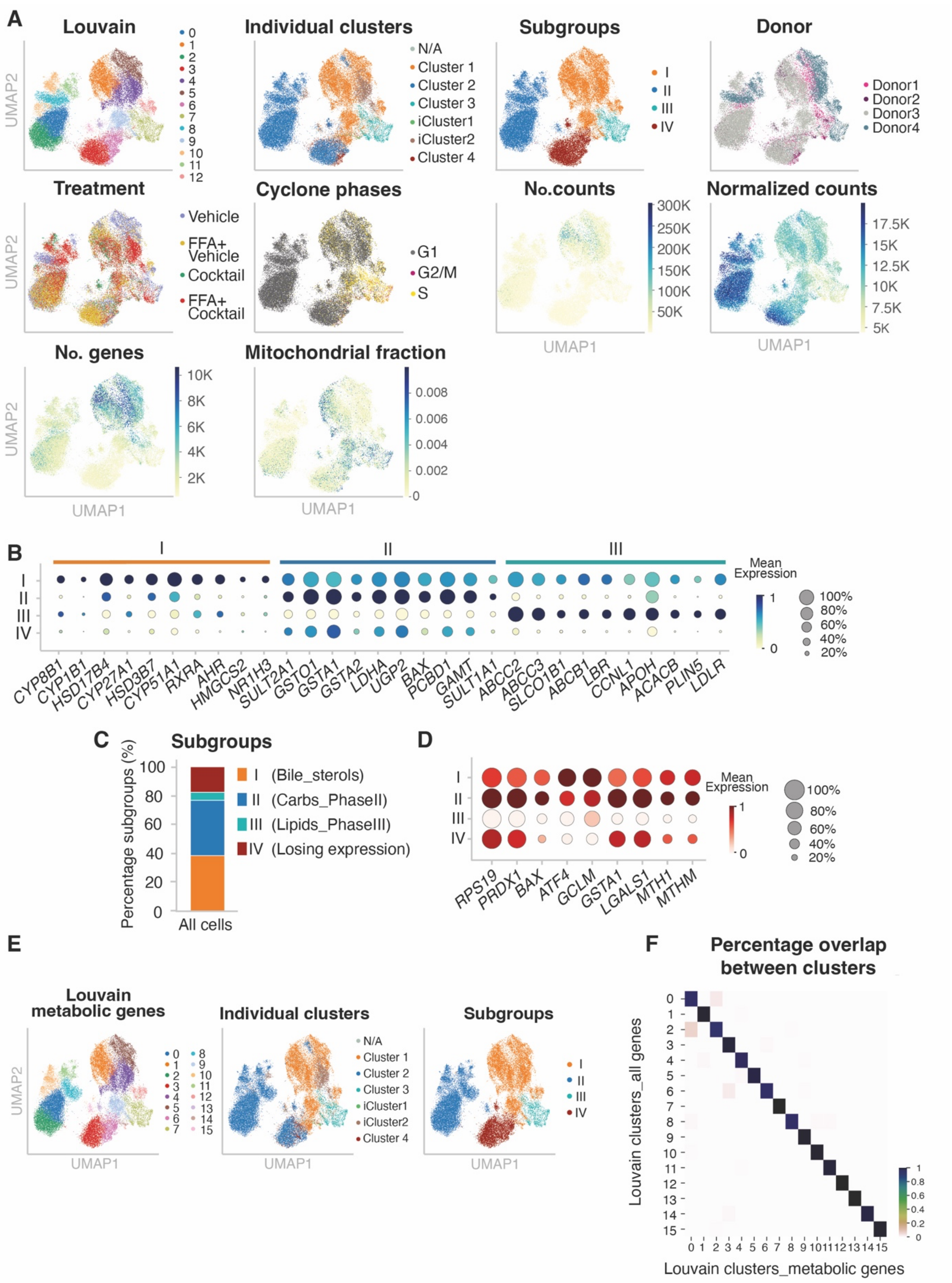

Figure S2: Clustering and cell annotation. (A) UMAP coloured by *Louvain* clusters, clusters identified in the individual donors, subgroups, donor, treatment, cell cycle phases identified by

cyclone, number of counts per cell, normalized counts per cell, number of genes per cell, and percentage of mitochondrial genes per cell. **(B)** Dot plot highlighting 10 representative marker genes in different metabolic categories per functional subgroup (color corresponding to scaled mean expression, dot size corresponding to percentage of cells expressing gene) **(C)** Bar plot depicting the percentage of cells in each subgroup. **(D)** Dot plot showing stress-related marker genes in the 4 subgroups (color corresponding to scaled mean expression, dot size corresponding to percentage of cells expressing gene). **(E)** UMAP calculated on 1,757 metabolic marker genes alone, coloured by *Louvain* clusters, clusters identified in the individual donors, and subgroups. **(F)** Heatmap showing the percentage of cells assigned to the same *Louvain* clusters when comparing the analysis based on all genes (A) and on the metabolic marker genes alone (D).

Figure S3

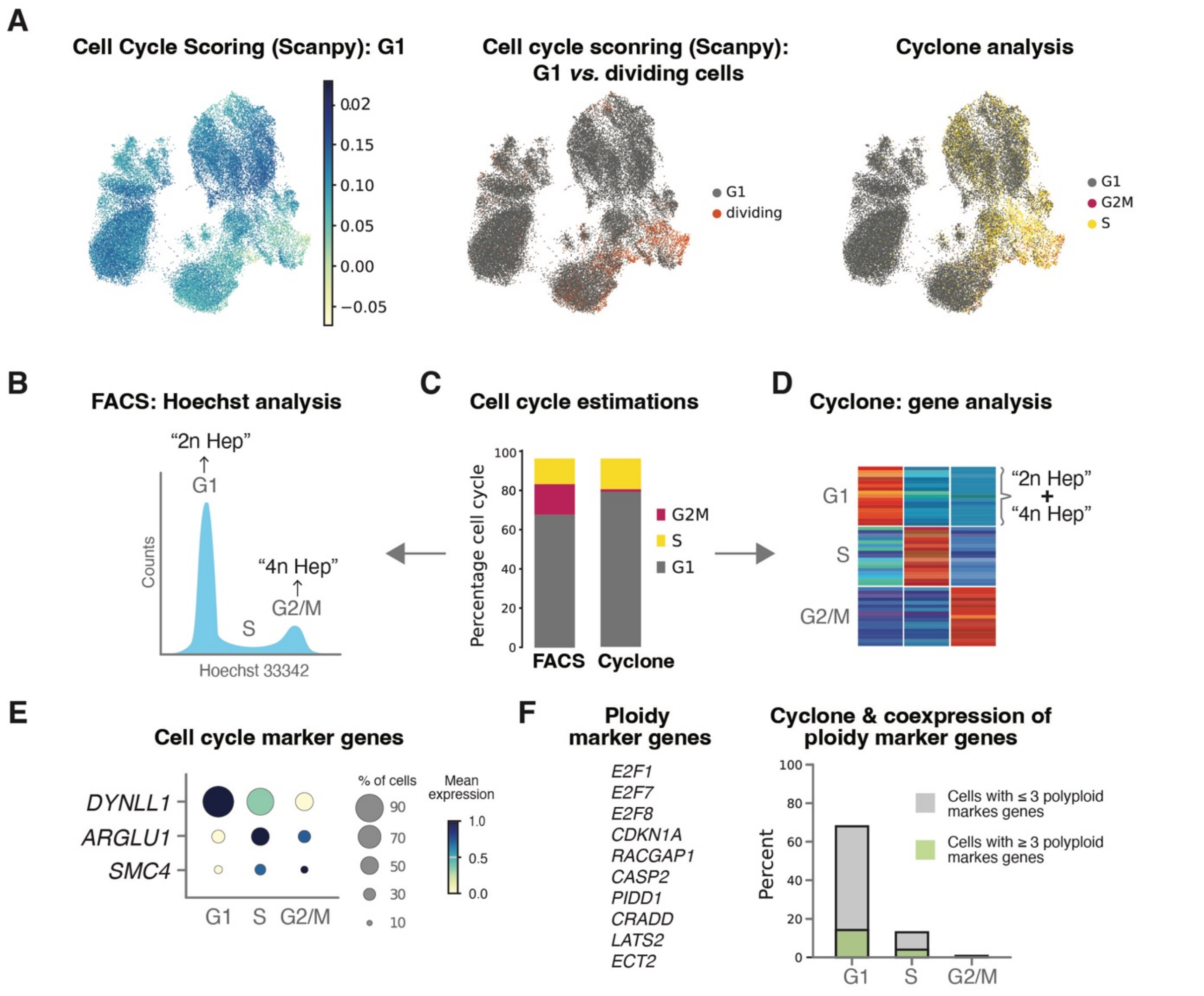

Figure S3: Comparison between cell cycle analysis using FACS and Cyclone. (A) Scoring of cells based on expression of G1 marker genes shows comparable results to Cyclone analysis. **(B)** Illustrative representation (cartoon) of tetraploid hepatocytes identified in G2/M phase using FACS analysis and Hoechst. **(C)** Percentage of Cell cycle estimations between FACS and Cyclone analysis. **(D)** Illustrative representation (cartoon) of Cyclone and cell cycle markers genes identifying diploid and tetraploid hepatocytes in G1/G0. **(E)** Dot plot of cell cycle marker genes showing that primary hepatocytes are mainly in G1/G0 phase as expected. **(F)** Percentage of hepatocytes with 3 or more polyploid marker genes co-expressed in different cell cycle stages showing that potential tetraploid hepatocytes are capture in G1 phase using Cyclone.

Figure S4

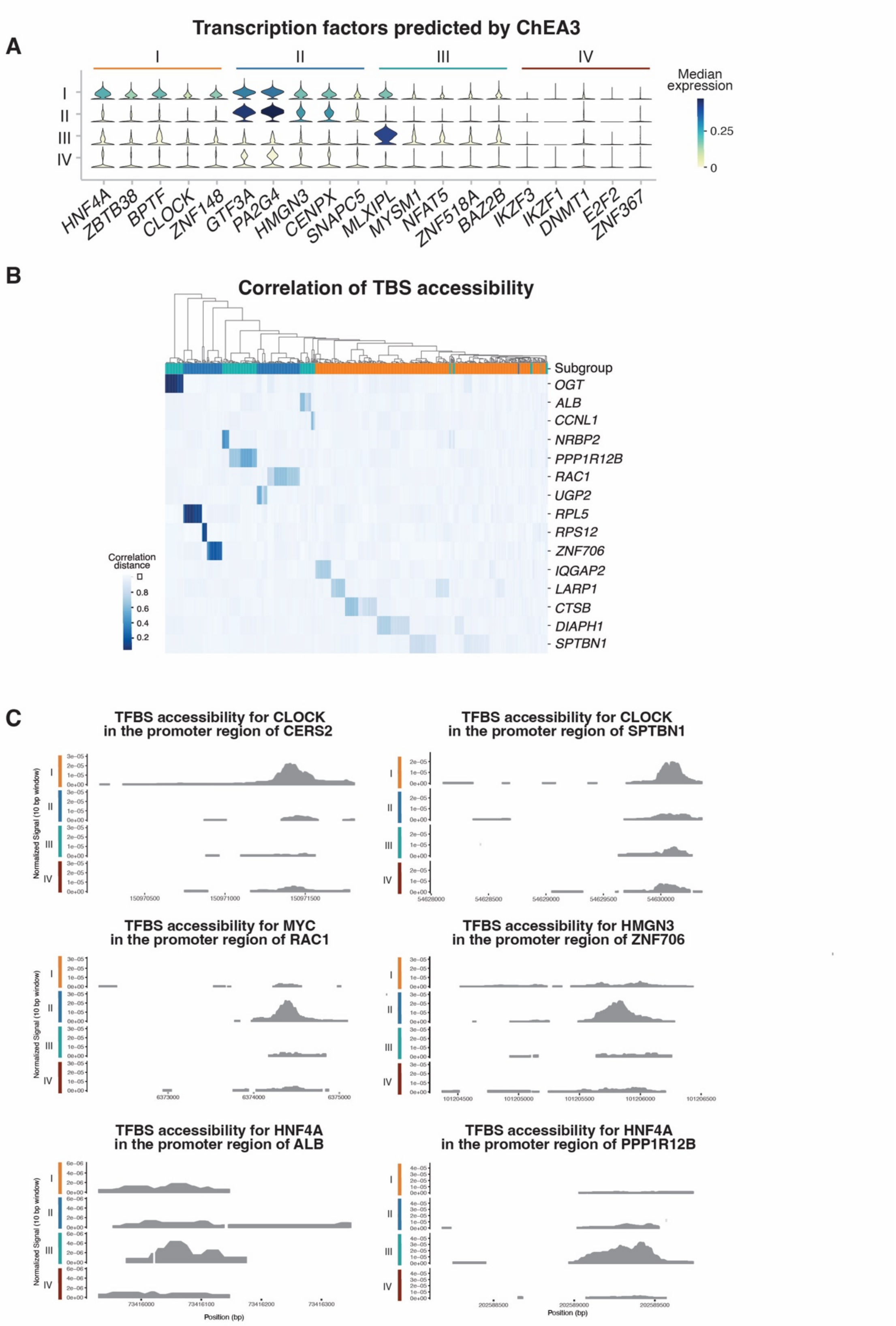

Figure S4: ChEA3 and scATAC-seq analysis. (A) Stacked violin plots showing 5 of the top 25 predicted transcription factors per subgroup regulating functional differences between the subgroups in DMSO. **(B)** Heatmap clustering the TFBS (columns) of subgroup-specific DEGs

(rows) based on their openness correlation. TFBS are colored based on the subgroup-specific DEG in their proximity (orange: subgroup I, blue: subgroup II, turquoise: subgroup III). **(C)** Track plots showing the TFBS region of ChEA3-predicted TFs upstream of two representative DEGs per subgroup; for subgroup I, CERS2 and SPTBN1; for subgroup II, RAC1 and ZNF706; and for subgroup III, ALB and PPP1R12B .

Figure S5

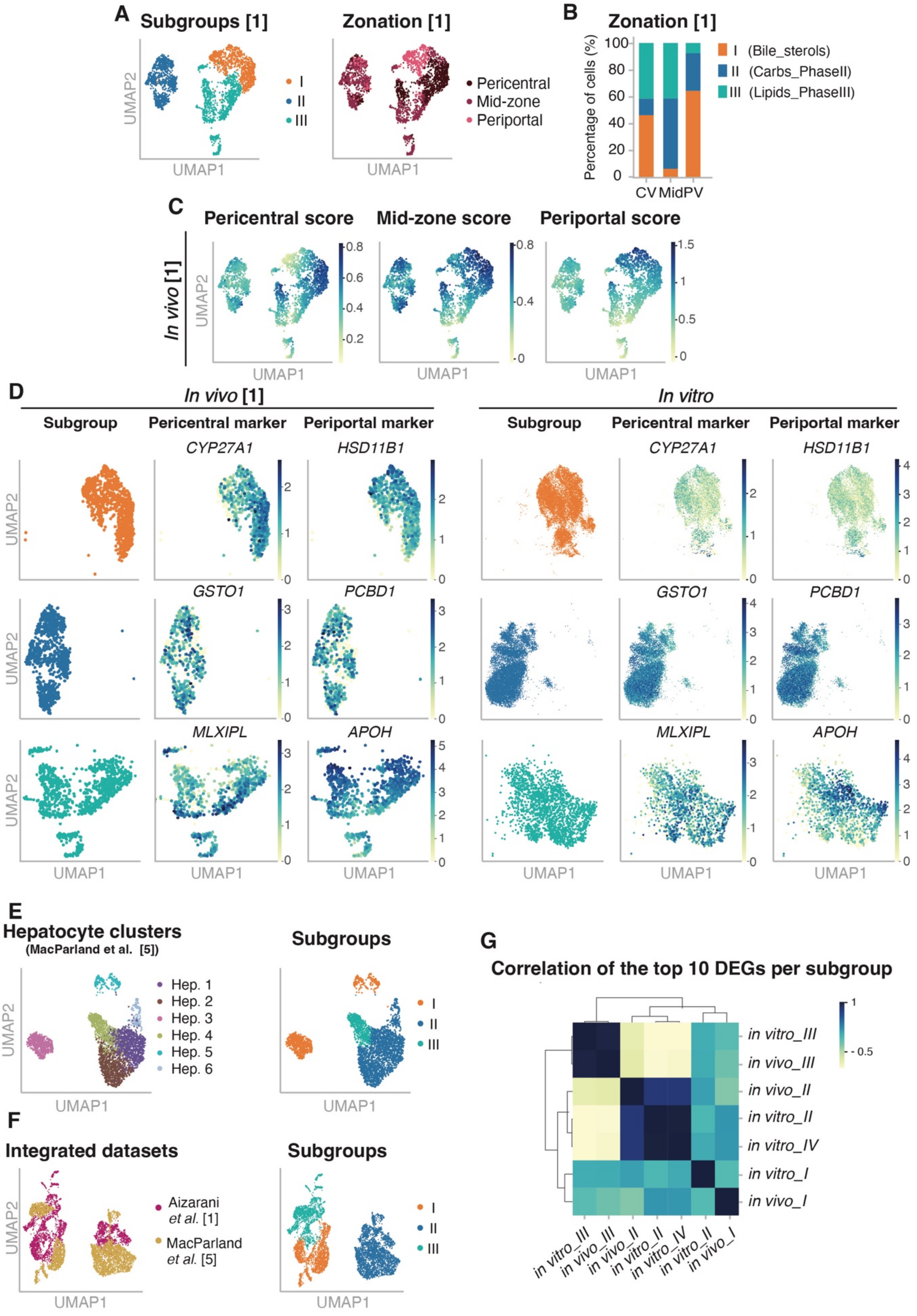

A
Subgroups [1]
Zonation [1]
I
II
III
Pericentral
Mid-zone
Periportal
UMAP1
UMAP2
B
Zonation [1]
Percentage of cells (%)
CV
Mid
PV
I (Bile_sterols)
II (Carbs_PhaseII)
III (Lipids_PhaseIII)
C
In vivo [1]
Pericentral score
Mid-zone score
Periportal score
D
In vivo [1]
In vitro
Subgroup
Pericentral marker
Periportal marker
CYP27A1
HSD11B1
GSTO1
PCBD1
MLXIPL
APOH
E
Hepatocyte clusters
(MacParland et al. [5])
Hep. 1
Hep. 2
Hep. 3
Hep. 4
Hep. 5
Hep. 6
Subgroups
F
Integrated datasets
Aizarani et al. [1]
MacParland et al. [5]
Subgroups
G
Correlation of the top 10 DEGs per subgroup
in vitro_III
in vivo_III
in vivo_II
in vitro_II
in vitro_IV
in vitro_I
in vivo_I

Figure S5: *In vitro* and *in vivo* comparisons of subgroups of hepatocytes. (A) UMAPs of the *in vivo* data from Aizarani *et al*. 2019 colored by hepatocyte subgroup (left) and their assigned zone along the pericentral-periportal axis (right). **(B)** Bar plot showing the percentage of cells in each subgroup assigned to each of the three lobule zones: pericentral, mid-zone and periportal. **(C)** UMAPs of the *in vivo* data from Aizarani *et al.* 2019 depicting the scores calculated for the pericentral, mid- and periportal zone, respectively. **(D)** UMAPs visualizing the expression of pericentral and periportal marker genes *in vivo* (Aizarani *et al.* 2019, left) and *in vitro* (right) in each of the three metabolically active subgroups. **(E)** UMAPs showing the embedding of the *in vivo* dataset from MacParland *et al.*[5] colored by their identified hepatocyte clusters (left) and the annotated subgroups based on marker gene expression (right). **(F)** UMAPs depicting the integrated embedding of the two *in vivo* datasets[1][5] colored by dataset (left) and annotated subgroups (right). **(G)** Cluster map showing the correlation between the top 10 DEGs per subgroup *in vitro* and *in vivo*.

Figure S6

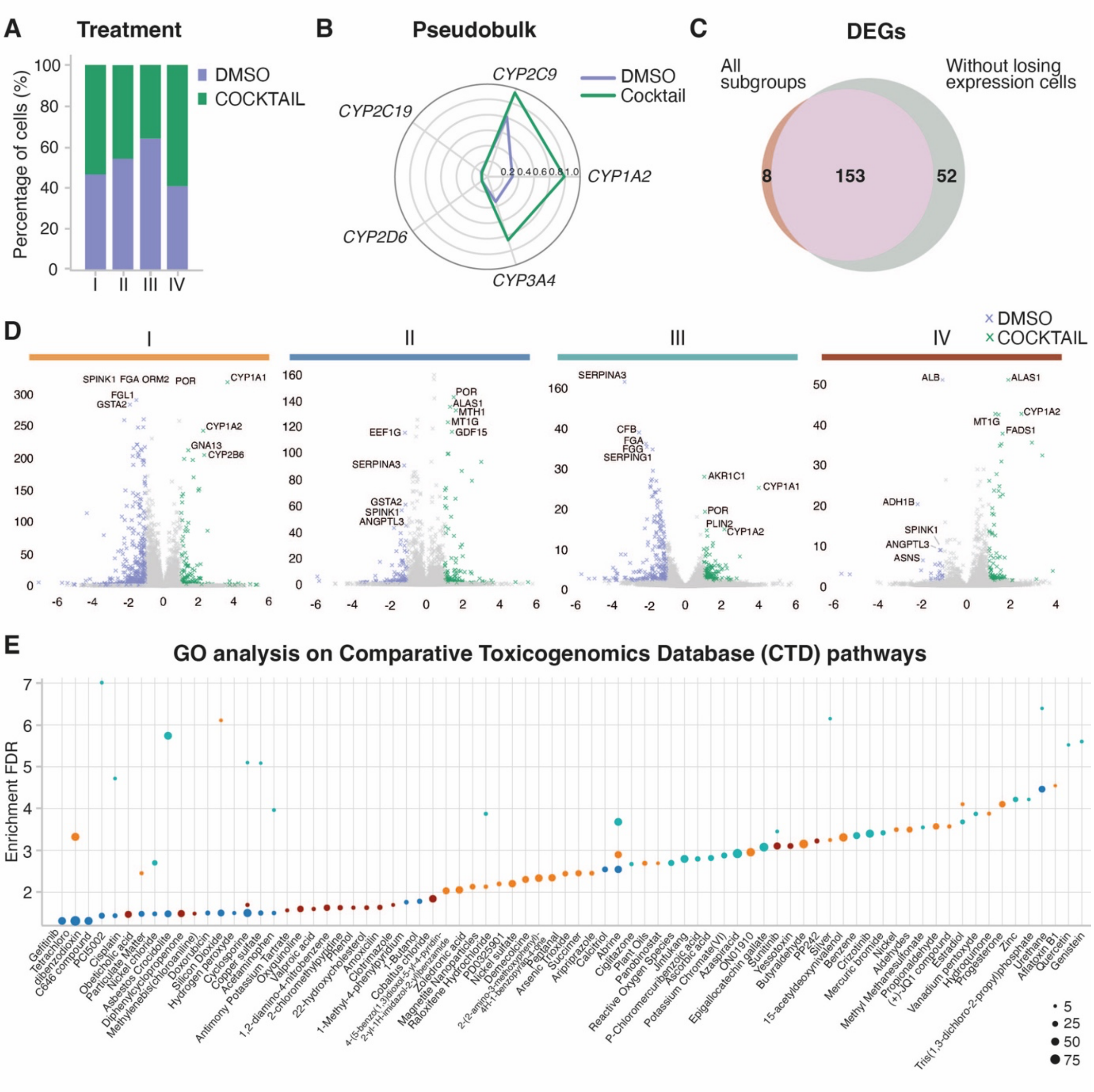

Figure S6: Transcriptional signatures in response to the phenotyping cocktail. (A) Bar plot showing the percentage of cells treated with and without cocktail in every subgroup. **(B)** Radar chart depicting expression levels of the five inducible cytochromes upon DMSO (purple) and Cocktail (green) treatment. **(C)** Venn diagram depicting the overlap of DEGs detected with (red) and without (grey) subgroup IV (losing expression). **(D)** Volcano plot showing differential gene expression in each subgroup for Cocktail- vs. DMSO- treated cells. **(E)** Scatter plot depicting enrichment of the genes specifically up-regulated in each of the 4 subgroups in pathways known to be involved in the metabolism of given chemical compounds (Drug.CTD database). The size of the dot corresponds to the number of overlapping genes in a given pathway.

Figure S7

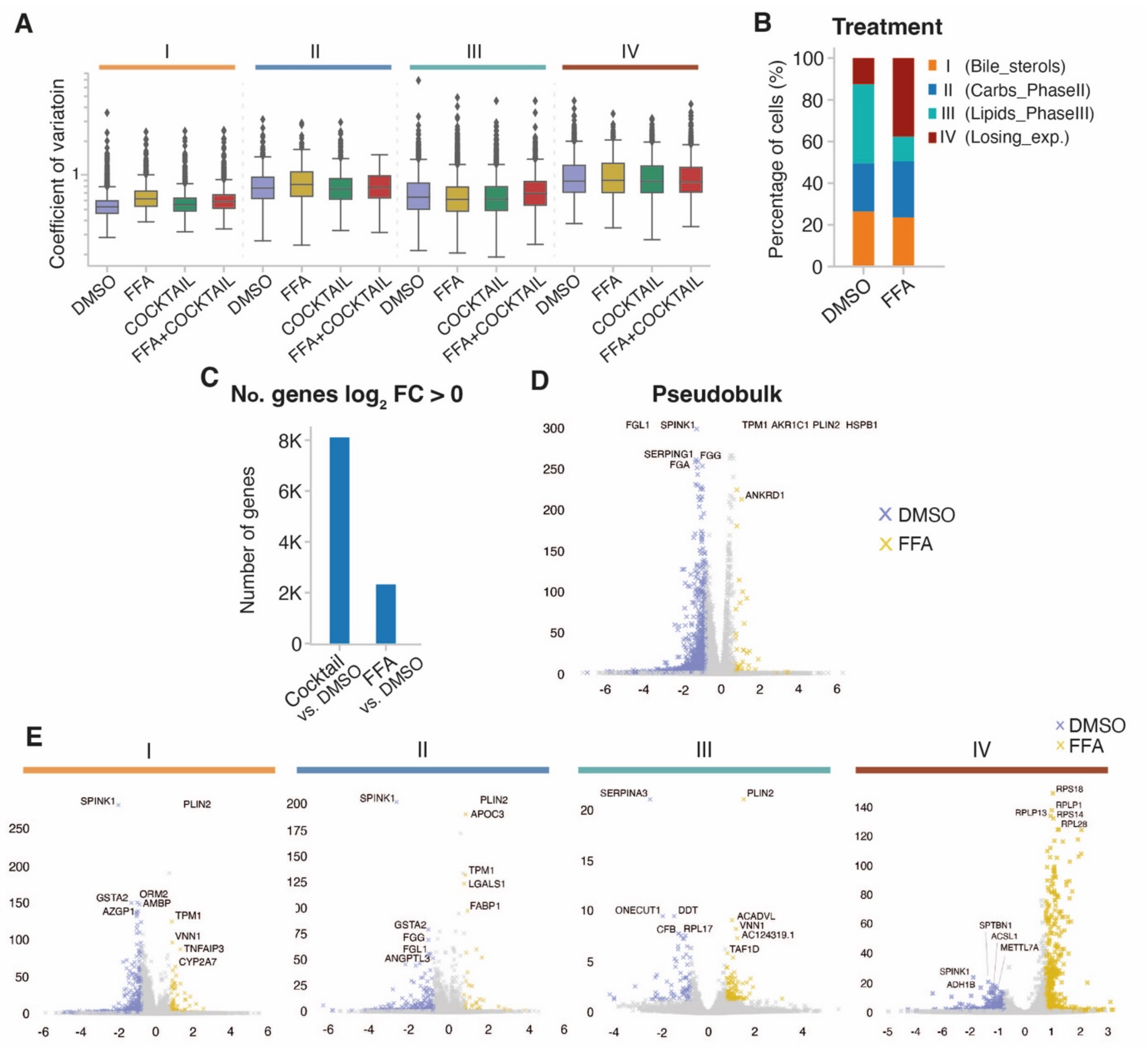

Figure S7: Transcriptional profiles upon intracellular lipid accumulation. (A) Box plots depicting the coefficient of variation in every treatment condition per subgroup. **(B)** Bar plot showing the percentage of cells from each subgroup in DMSO and FFA treatment condition. **(C)** Bar plot representing the number of genes with positive log_2-fold change towards DMSO level in Cocktail vs. DMSO and FFA vs. DMSO treatment conditions. **(D)** Volcano plot showing differential gene expression for FFA- vs. DMSO- treated cells in pseudobulk. **(E)** Volcano plot showing differential gene expression in each subgroup for FFA- vs. DMSO-treated cells.

Figure S8

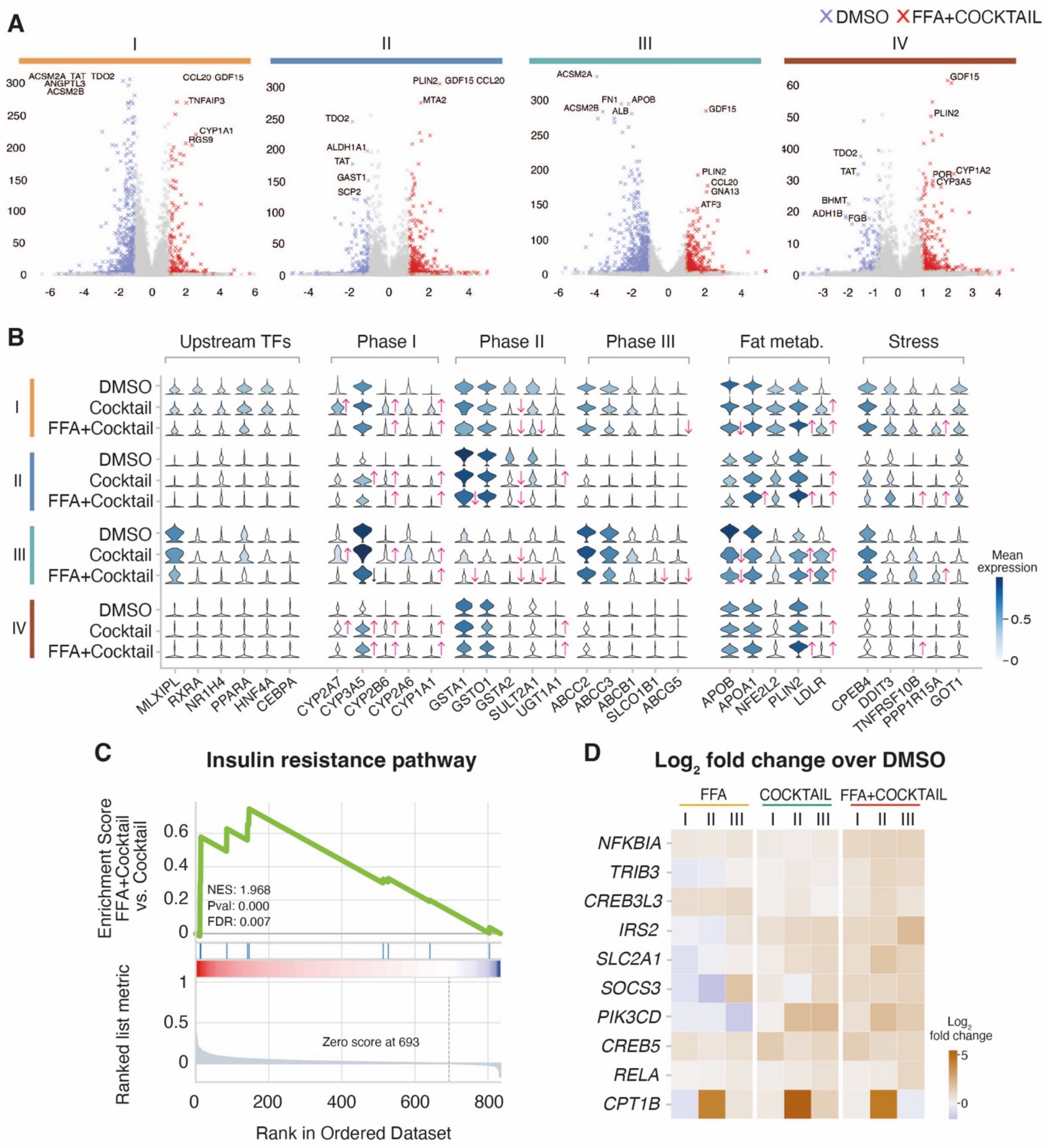

Figure S8: Transcriptional dysregulation of multiple metabolic pathways upon fat accumulation. (A) Volcano plot showing differential gene expression for FFA+Cocktail- vs. DMSO-treated cells per subgroup. **(B)** Stacked violin plots showing transcription factors upstream of the cytochrome P450 family, members of the cytochrome P450 family, phase II enzymes, phase III enzymes, markers of lipid metabolism, and markers of stress in DMSO-treated cells and cells treated with cocktail, and FFA+Cocktail per functional subgroup (↑: indicates up-regulation towards DMSO; ↓: indicates down-regulation towards DMSO, t-test). **(C)** Gene Set Enrichment Analysis (GSEA) plot for FFA+Cocktail vs. Cocktail-treated cells on the pathway of “Insulin resistance”, enriched in the FFA+Cocktail vs. DMSO-specific genes. **(D)** Heatmap depicting log_2-fold change to DMSO-level of genes involved in the insulin resistance pathway that were enriched in FFA+Cocktail vs. Cocktail.

Figure S9

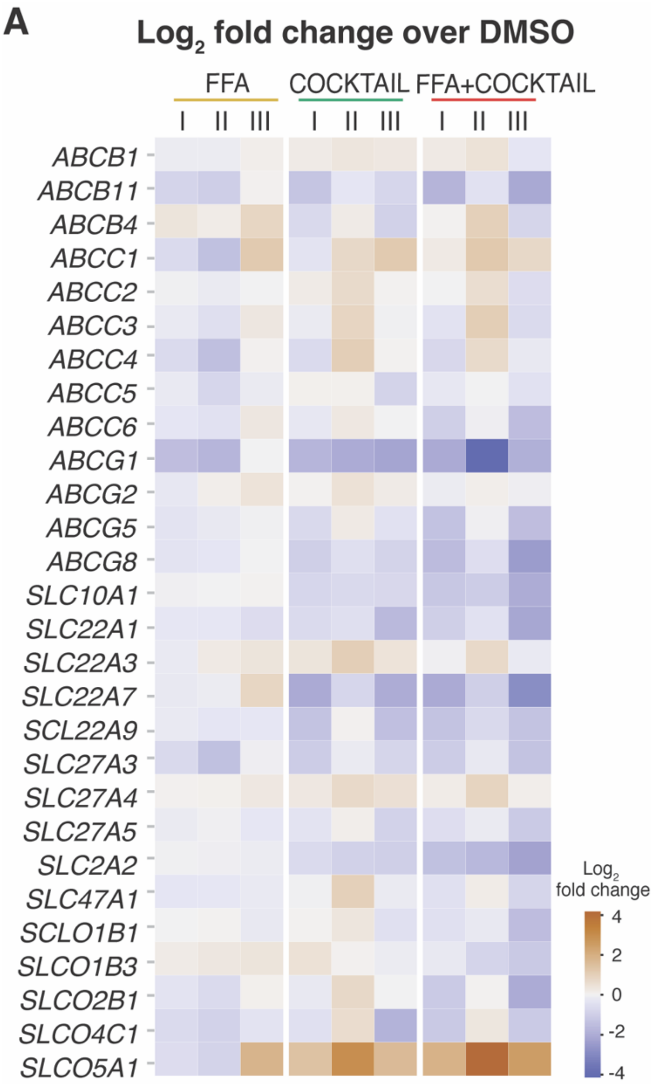

Figure S9: Transcriptomic changes on phase III transporter genes. (A) Heatmap depicting log_2-fold change to DMSO-level of 28 solute carriers and influx transporters in FFA, Cocktail, and FFA+Cocktail per subgroup.
