# Supplementary material for: Single-cell metabolic profiling reveals subgroups of primary human hepatocytes with heterogeneous responses to drug challenge
Source: Genome Biol. 2023 Oct 17;24:234. doi: 10.1186/s13059-023-03075-9 (PMC10583437; doi:10.1186/s13059-023-03075-9)
Supplement: Supplementary file 12 — Additional file 12. Review history. [file 13059_2023_3075_MOESM12_ESM.docx]

Review History

**First round of review**

**Reviewer 1**

**Were you able to assess all statistics in the manuscript, including the appropriateness of statistical tests used?**

No, I do not feel adequately qualified to assess the statistics.

**Comments to author:**

In this manuscript, the authors use single cell sequencing to determine the heterogeneity of human hepatocytes in terms of their metabolism. The aim of the study is good. They find that hepatocytes can be subgrouped into 4 groups, one of which was known before. Based on this subgrouping, the authors try to decipher the functions of these hepatocytes.

Although this is an interesting study, I have to admit (with sincere apologies) that I did not enjoy reading this manuscript since the data is scant and the authors (mostly using bioinformatic approaches) are trying to conclude findings without confirming them. I am afraid that the data does not support most of their claims.
Overall, the data is very limited and I cannot see how to support the claims that are being made. In addition, since the authors want to investigate change in metabolism of individual hepatocytes, I am not sure that single cell sequencing is an adequate approach without confirming any metabolites. Therefore, this is study is only based on mRNA expression which does not necessarily reflect metabolism in hepatocytes (without using other approaches).

Major points:
1. The manuscript contains 11472 pages: can any human process that many pages? It would be beneficial to limit the manuscript to approximately 50 pages or less…
2. The authors use commercially available hepatocytes from Lonza without any validation. They have no control over how these hepatocytes have been treated/collected. At least, the authors should mention or discuss this limitation in detail.
3. Figure 1D is hard to understand. If all percentages are added together, this results in approximately 1% of the genes that are expressed in any type of hepatocytes. Does this mean that 99% of genes are not expressed in any type of hepatocyte?
4. Figure 1E: the authors mention that they used the tool "cyclone" to determine the cell cycle phase. Have they validated their data using other approaches? I just wonder what this means? Group I, II, and IV seem to be ok but group III is very different. Why is that so and can this be seen in vivo?
5. Line 200-210: this is a good example why this manuscript is hard to read. The authors use ChEA3, a ChIP-enrichment analysis tool, to predict transcription factors differentially expressed in subpopulations of hepatocytes. Nevertheless, there is no follow up or validation. ChEA3 without validation (ChIP-PCR or ChIP-seq) bears little value and it is just a prediction.
6. Line 218-244: the authors claim to have done "in vivo" analysis. Nevertheless, what they really have done is to compare their data to published data. This does not bear much value. Like all other researchers, the authors should do "real" in vivo experiments to confirm and validate their predictions.
7. Figure 2A: why is there an upregulation of CYP2C9 in the DMSO treated subgroup III?
8. Paragraph around line 340: it is difficult to understand what message the authors want to convey and on what data this is based on.
9. The discussion is quite vague and it is hard to understand what is the central message. The authors should include an honest assessment of the limitations of this study.

**Reviewer 2**

**Were you able to assess all statistics in the manuscript, including the appropriateness of statistical tests used?**

Yes, and I have assessed the statistics in my report.

**Comments to author:**

The manuscript by Sanchez-Quant et al presents very interesting results on single-cell RNA seq analysis of primary human hepatocytes and how lipid stimulation affects their transcriptional programs and drug metabolism.

Overall the manuscript is very well written, focuses on the topic which of high interest and provides novel insights.

I have a few comments that, if addressed, may improve the manuscript.

First, there is an observable heterogeneity between the donors. Please show that each donor contributes to each of the hepatocytes subgroup (as expected if this would be groups associated with cell cycle phases or zonation), or please explain if it's not the case.

Second, please consider doing UMAP and clustering analysis only for metabolic genes. This is in particular necessary since you use the title "single-cell metabolic" profiling.

Third, do you have any evidence that lipid accumulation (or stimulation) affects drug metabolism and not drug uptake?

**Authors Response**

**Point-by-point responses to the reviewers’ comments:**

**Reviewer #1:**

In this manuscript, the authors use single cell sequencing to determine the heterogeneity of human hepatocytes in terms of their metabolism. The aim of the study is good. They find that hepatocytes can be subgrouped into 4 groups, one of which was known before. Based on this subgrouping, the authors try to decipher the functions of these hepatocytes.

Although this is an interesting study, I have to admit (with sincere apologies) that I did not enjoy reading this manuscript since the data is scant and the authors (mostly using bioinformatic approaches) are trying to conclude findings without confirming them. I am afraid that the data does not support most of their claims.

Overall, the data is very limited and I cannot see how to support the claims that are being made. In addition, since the authors want to investigate change in metabolism of individual hepatocytes, I am not sure that single cell sequencing is an adequate approach without confirming any metabolites. Therefore, this is study is only based on mRNA expression which does not necessarily reflect metabolism in hepatocytes (without using other approaches).

Major points:

1) **The manuscript contains 11472 pages: can any human process that many pages? It would be beneficial to limit the manuscript to approximately 50 pages or less...**

We do apologize for the formatting mistake, which occurred during the document uploading process. The supplementary material was added to the main text as “tables” during the manuscript submission. We encountered technical issues during the online submission and we could not proofread the final pdf. We have corrected the submission and we do apologize for the inconvenience.

2) **The authors use commercially available hepatocytes from Lonza without any validation. They have no control over how these hepatocytes have been treated/collected. At least, the authors should mention or discuss this limitation in detail.**

We do appreciate the Reviwer#1’s comment and we totally agree with it. For that reason, we only used certified hepatocytes by the supplier (Lonza). More specifically, we purchased human hepatocytes, cryopreserved, palatable and interaction qualified (ref. #HUCPI, Lonza). These hepatocytes are characterized for three major drug-drug interaction: transporter activity, enzyme activity and enzyme gene induction potential. In our revised manuscript, we have added all these additional information in a supplementary table 1 and Method section respectively.
For instance, enzymatic activity and mRNA induction levels were measured for CYP1A2, CYP2B6 and CYP3A4. Based on Lonza’s reports (Certificate of Analysis are now included in Supp Table 1), the cells used in our experiments were metabolically competent regarding their phase I and phase II metabolism.
Additionally, we have further compared the basal mRNA levels of *CYP1A2* in our dataset and the induced enzymatic activity assessed by Lonza. As expected, we found a drug-induced upregulation of the mRNA levels by caffeine (Sanchez-Quant et al.) and induced enzymatic activity by omeprazole (Lonza, Certificate of analysis) (Figure 1 for Reviewers), showing that the purchased PHH are suitable for drug-induction studies.

***Figure 1 for Reviewers.*** *CYP1A2 fold induction of mRNA and enzymatic activity after incubation with caffeine and omeprazole respectively. Primary Human Hepatocytes from 4 different donor were used.*

In the revised manuscript, we have clarified the source of hepatocytes in the method section: *>> Briefly, commercially plateable and interaction-qualified cryopreserved human hepatocytes (Lonza, Walkersville, MD, USA) were purchased from Lonza from four different donors: <<*

**3) Figure 1D is hard to understand. If all percentages are added together, this results in approximately 1% of the genes that are expressed in any type of hepatocytes. Does this mean that 99% of genes are not expressed in any type of hepatocyte?**

We appreciate the reviewer’s comment and we have corrected the scale of the y-axis. The y-axis was labeled incorrectly as the scale was going from 0 to 1 when the label indicated the values were in %. We have corrected this issue by adjusting the scale to 0-100 to be consistent with the neighboring panel. To increase the clarity, we have also re-label the y-axis as “Percentage of cells per gene (%)”.

The Figure 1D shows how a given gene is expressed on different number of cells depending on the hepatocyte subgroup. Due to the sparsity of scRNA-seq, this number is on average below 40% in our data. This does not mean that 99% of genes are not expressed anywhere, but it means that an individual gene is usually expressed in a low number of cells (and in even fewer cells for the losing expression group, subgroup IV).
To illustrate this, we have now included a bar plot for *CYP1A2* and *CYP3A4*, showing the percentage of cells expressing those genes in each subgroup respectively. (Supplementary Figure 1E). In other words, *CYP1A2* is expressed in 50% of the cells in subgroup I, and 30% of the cells. *CYP3A4* is expressed in a similar number of cells in subgroup II. Overall, these results indicate that genes are expressed in a lower percentage of cells in subgroup IV compared to the other metabolically competent subgroups.

***New figure in revised manuscript.***

***Supp. Figure 1E*** *showing the percentage of cells expressing CYP1A2 and CYP3A4 respectively.*

**4) Figure 1E: the authors mention that they used the tool "cyclone" to determine the cell cycle phase. Have they validated their data using other approaches? I just wonder what this means? Group I, II, and IV seem to be ok but group III is very different. Why is that so and can this be seen in vivo?**

We appreciate the Reviewer’s suggestion and we have added additional information about the cyclone tool in our revised manuscript, and performed validation experiments. Conventionally, cell cycle analysis is estimated by the quantitation of DNA content using flow cytometry. Using DNA binding dyes, this method assumes that dyes are bound stoichiometrically, and therefore proportionally to the amount of DNA present in the cells. In this way cells that are in S-phase will have more DNA than cells in G1/G0. Cyclone uses the single-cell transcriptome to allocate cells to their cell-cycle stage on the basis of the expression of specific cell-cycle marker genes [1]. This method has been extensively used (more than 384 citation) and experimentally validated by the authors (Scialdone *et al* [1]) also using primary human hepatocytes. Following the Reviewer’s suggestion, we have also performed convectional Flow Cytometry analysis using Hoechst in PHH from two additional human donors (Figure 2 for Reviewers).

***Figure 2 for reviewers.*** *Validation and comparison of cell cycle analysis. A) Cell cycle analysis using Hoechst in PHH from two additional human donors. B) Comparison of the percentage of cells assigned to different cell cycle stages based on FACS or Cyclone analysis FACS (% of parental gating) or Cyclone analysis (%of cell cycle).*

From these FACS results we found that, on average, 11.8% of PHH are in S-phase and 13.9% in G2M phase. This roughly matches the predictions from cyclone, for which overall 17.6% of cells were found in S-phase. It has been reported that PHH can expand *in vitro* under certain experimental conditions but these hepatocytes are called bi-phenotypic, partially retaining hepatic features while gaining expression of progenitor-associated genes [2]. Therefore, it is possible that some hepatocytes initiate the process of cell division, expressing specific cell-cycle marker genes. Thus, these dividing hepatocytes would be assigned to S-phase by Cyclone, while the same hepatocytes will not be assigned to S-phase by FACS analysis until the entire genome is duplicated (explaining the higher percentage of cells in S-phase by Cyclone compared to FACS, Figure for reviewers 2B). We believe that Cyclone has higher accuracy in assigning cell-cycle stages based on changes in gene expression of cell-cycle markers genes, while FACS only detect unspecific duplication of the genome using Hoechst staining.

Additionally, to show the differences in dividing cells between subgroups, we also performed Cyclone analysis on the integrated *in vivo* dataset [3] [4], consisting of in total 14 human donors. *In vitro*, we found that 84.6% of cells in subgroup III were in either S-phase (73.2%) or G2M-phase (11.4%). In the publicly available *in vivo* data set, the cell cycle analysis also showed the highest proportion (87%) of dividing cells in subgroup III with 86.3% in S-phase, and 0.7% in G2M. In summary, both FACS and computational analysis show similar percentage of cells assigned to S-phase. Importantly, the proportion of dividing cells differs between hepatocyte subgroups and these differences are also found *in vivo*.

***Figure 3 for reviewers.*** *Cyclone analysis on the integrated in vivo dataset [3,4], consisting of in total 14 human donors*

*In vivo*, it is well-known that after two-third partial hepatectomy, there is a prominent hepatic fat accumulation in the first round of DNA synthesis before mitotic activity [5] [6]. During partial hepatectomy-induced regeneration, four continuous waves of hepatic replication have been described associated to a tight control on the number of hepatocytes entering into cell cycle. Interestingly, three waves of hepatic fat accumulation were coupled to hepatocyte replication [6], suggesting that lipid accumulation and cell cycle in hepatocytes are co-regulated processes. However, the precise molecular mechanism underlaying changes in cell cycle and fat accumulation still remain unknown [7]. This topic has been included in the discussion section.

Line 974

>> In fact, it is well-known that after two-third partial hepatectomy, there is a prominent hepatic fat accumulation in the first round of DNA synthesis before mitotic activity [5, 8]. During partial hepatectomy-induced regeneration, four continuous waves of hepatic replication have been described associated to a tight control on the number of hepatocytes entering into cell cycle. Interestingly, three waves of hepatic fat accumulation were coupled to hepatocyte replication [6], suggesting that lipid accumulation and cell cycle in hepatocytes are co regulated processes. However, the precise molecular mechanism underlaying changes in cell cycle and fat accumulation still remain unknown [7].<<

**5) Line 200-210: this is a good example why this manuscript is hard to read. The authors use ChEA3, a ChIP-enrichment analysis tool, to predict transcription factors differentially expressed in subpopulations of hepatocytes. Nevertheless, there is no follow up or validation. ChEA3 without validation (ChIP-PCR or ChIP-seq) bears little value and it is just a prediction.**

We would like to thank the Reviewer’s suggestions to improve the clarity and impact of our manuscript. We performed ChEA3 analysis to explore the regulatory networks that might be governing the observed transcriptional changes in the different hepatocyte subgroups. As the Reviewer1 indicates, ChEA3 is a transcription factor enrichment analysis tool that ranks the transcriptional factors that have been shown to be associated to specific genes by different experimental approaches (e.g. by Chip-seq or Co-IP). With the expansion of NGS, the increasing numbers of publicly available databases employed by ChEA3 and other similar tools lead to more reliable predictions.

For the experimental validation of our results, traditional ChIP-seq or ChIP-PCR measurements on hepatocytes in bulk will not capture the cellular heterogeneity between subgroups. Therefore, to circumvent this problem, we have performed single-cell ATAC-seq on DMSO-treated cells in one human donor. The aim was to validate whether the predicted transcription factors and corresponding cis-regulatory elements are accessible at the genomic level in the different hepatocyte subgroups.

Although at the genomic level, we did not observe clear clusters of hepatocyte subgroups, we do observe heterogeneity in the chromatin openness of the predicted TFBS regions. Moreover, we measured co-accessibility of regions to investigate whether cells share the same chromatin landscape for the regions of interest (Supplementary figure 3). Indeed, we found that the binding sites of the predicted transcription factors at the marker gene regions were open in the same cells. For instance, the transcription binding site (TFBS) of CLOCK at the marker genes CERS2 and SPTBN1 were co-accessible in the same cells, suggesting a chromatin profile of subgroup I cells. This indicates that the heterogeneity among PHH detected in scRNA-seq is measurable at the chromatin level and is reflected by the results obtained in the ChEA3 analysis.

***New figure in revised manuscript.***

***Supp. Figure* 3** *Single cell ATAC-seq on primary human hepatocytes incubated with DMSO shows co-accessible*

*genomic regions for the transcription factors predicted by ChEA3.*

It is worth to mention that the predicted transcription factors were found to be differentially expressed between hepatocytes subgroups, suggesting that ChEA3 successfully predicted relevant upstream regulators that are expressed in the cell and with accessible cis-regulatory elements.

We have included the new scATAC-seq analysis results in Supplementary figure 3. Moreover, we have expanded the method section to explain how these data were obtained and analyzed (lines 1310).

**6) Line 218-244: the authors claim to have done "in vivo" analysis. Nevertheless, what they really have done is to compare their data to published data. This does not bear much value. Like all other researchers, the authors should do "real" in vivo experiments to confirm and validate their predictions.**

Following the Reviewers’ comment, we have rephrased the revised manuscript in several locations to clarify how the comparison between *in vitro* and *in vivo* data sets was performed and which publicly available data was used.

Remarkably, by using publicly available data, we are able to compare our *in vitro* primary human hepatocytes with livers from a cohort of 14 human donors, a number that is challenging to achieve for two main reasons: i) restrictions to access human samples, and ii) costs of single-cell genomics per sample. Human cell atlas initiatives show a great collective effort to provide information about cellular composition from human healthy tissues and disease conditions.

Therefore, our revised manuscript clearly specifies how many human livers were analyzed. In our first manuscript, we included the analysis from the data generated by Aizarani *et al.* (9 donors), and by MacParland *et al.* (5 donors). In our revised manuscript, we have included the analysis of these two data sets separately, showing that using scRNA-seq data from 5 human livers (MacParland *et al.),* all the hepatocyte subgroups could also be identified (Supp. Figure 4E-G). Accordingly, we have rephrased our manuscripts and have added a new supplementary Figure 4E-G and Method description.

***New figure in revised manuscript.***

***Supp. Figure* 4** *Identification of hepatocytes subgroups in 14 human donors from publicly available data sets.*

***Figure 2A*** *Violin plots depicting expression levels between Cocktail and DMSO of the five CYPs involved in the metabolism of the five-drug cocktail in pseudobulk (left), and in each hepatocyte subgroup (right)*

**7) Figure 2A: why is there an upregulation of CYP2C9 in the DMSO treated subgroup III?**

Figure 2A shows the basal expression levels of the five targeted CYPs (i.e. *CYP2D6, CYP2C19, CYP2C9, CYP3A4, CYP1A2*) in primary human hepatocytes incubated with DMSO (left). Our basal expression levels are in agreement with the previously reported race-dependent differences in constitutive cytochrome P450 mRNA and activity levels [9]. More specifically, Liu et al showed the differential expression levels of *CYP2C9* in African American, Caucasian and Hispanic donors. In agreement with Liu et al, we also observed an increased mRNA basal level of *CYP2C9* compared to *CYP1A2* and *CYP2D6* (Figure 4 for reviewers).

***Figure 4 for Reviewers****. Bar plots showing the mean expression of constitute levels of CYPs upon incubation with DMSO, A) per cytochrome, and B) per subgroup of hepatocytes.*

Our single cell analysis allows us to identify subgroups of hepatocytes with different constitutive or basal mRNA levels for different CYP P450 genes. For instance, *CYP2C9* shows a higher basal or constitutive expression level compared to other CYPs.

Figure 2A focuses on changes in mean gene expression (e.g., intensity of color) and gene expression distributions (e.g. shape of violin plots). However, in the Figure 5 for Reviewers, it is shown the mean expression of CYPs per cell (e.g. trackplot), showing that the mean expression of *CYP2C9* is higher in cells from subgroup III. Additionally, it is shown that the upregulation of *CYP2C9* expression in subgroup III after incubation with Cocktail, only occurred in a fewer number of cells. Therefore, subgroup III contributes with less cells to the average mean expression in the

pseudo-bulk. Our results shows that CYP’s basal transcription is higher in different subpopulations of hepatocytes, highlighting the transcriptional heterogeneity of *CYP2C9* expression among primary human hepatocytes.

***Figure 5 for reviewers.*** *Trackplot represents the distribution of gene expression across individual cells in the annotated clusters*

8) **Paragraph around line 340: it is difficult to understand what message the authors want to convey and on what data this is based on.**

Following the reviewers’ advice, we have edited this paragraph to improve the clarity and its take-home message.

In brief, the editions aimed to: First, we have clearly differentiated between differential basal transcriptional levels, and transcriptional upregulation upon cocktail treatment. Second, we have dissected the global transcriptional changes shared between subgroups, as well as the subgroup-specific changes. Third, scRNA-seq revealed subgroup-specific upregulated genes that were not detected in the pseudobulk studies.

Our take home message is that we found unexpected cellular heterogeneity present in a seemingly homogenous population of primary human hepatocytes which are classically considered the gold standard *in vitro* model for toxicology studies.

The last paragraph of the introduction has been re-written as follows, reinforcing the take-home message:

>>Here, we report concealed cellular heterogeneity in primary human hepatocytes which are classically considered a seemingly homogenous population of cells and a “gold standard” for *in vitro* hepatic assays. The comparison between pseudobulk and single-cell transcriptomics reveals four distinct hepatocyte subgroups that can be found also in fourteen human donors from publicly available data sets. We present evidence that chronic accumulation of lipids and xenobiotics lead to transcriptional variability and the impairment of multiple metabolic pathways in a subgroup-specific manner. Our results suggest that liver steatosis combined with multiple drug intake differentially affects the transcriptional profiles of individual cells decreasing the expression of key cytochrome P450 genes. <<

9) **The discussion is quite vague and it is hard to understand what is the central message. The authors should include an honest assessment of the limitations of this study.**

We have carefully reviewed the discussion section and rewritten the discussion in order to add clarity and state the limitations of our study.In summary, we have removed unnecessary details, highlight the technical limitations to dissect the role of hepatocytes subgroups *in vivo* due to the crosstalk between subgroup specialization and hepatocyte zonation, and clearly mentioned that further studies will be needed to understand the role of cellular heterogeneity on tissue function.

An example:

>>Another source of functional specialization is the well-known hepatic zonation in the liver. The zonation patterns found *in vivo* due to the oxygen and nutrient gradient along the lobule axis are not conserved in 2D *in vitro* models [10-15]. To deeply investigate if hepatocyte subgroups are related to reminiscent liver zonation, we have compared our findings *in vitro* with two publicly available *in vivo* data set comprising a cohort of 14 human donors [3, 4]. We have revealed the presence of the three metabolically active subgroups *in vivo*, mostly independent of zonation. Therefore, subgroup specialization and hepatocyte zonation might play both a key role in liver function. Since liver zonation has a profound impact in transcriptional regulation in individual cells [16], it is technically challenging to dissect the role of hepatocyte subgroups *in vivo*.<<

**Reviewer #2:**

The manuscript by Sanchez-Quant et al presents very interesting results on single-cell RNA seq analysis of primary human hepatocytes and how lipid stimulation affects their transcriptional programs and drug metabolism.

Overall the manuscript is very well written, focuses on the topic which of high interest and provides novel insights.

I have a few comments that, if addressed, may improve the manuscript.

**1) First, there is an observable heterogeneity between the donors. Please show that each donor contributes to each of the hepatocytes subgroup (as expected if this would be groups associated with cell cycle phases or zonation), or please explain if it's not the case.**

We thank the reviewer for this valuable comment. We have added a bar plot showing how each donor contributes to each hepatocyte subgroup (Supplementary figure 1D). As pointed out by the Reviwer2, we do observe that the proportion to which each donor contributes to each subgroup is variable. This is most likely explained by human inter-individual variability, such as ethnicity, tobacco, drug, and alcohol consumption, or metabolic capacity [9]. Human variability in regards the mRNA and enzymatic activity of CYP P450 based on gender, age and race has been recently shown by Liu et al [9]. In line with the comments of reviewer 1, we have extended supplementary table 1 to contain additional information on the donors provided by Lonza. Nevertheless, despite the variability present between donors, we show that each hepatocyte subgroup is present in each of the four donors.

***New figure in revised manuscript.***

***Supp. Figure* 1** Percentage of donors per hepatocytes subgroup (%)

**2) Second, please consider doing UMAP and clustering analysis only for metabolic**

**genes. This is in particular necessary since you use the title "single-cell metabolic"**

**profiling.**

We greatly appreciate this suggestion from the reviewer. We have now used 1,757 metabolic

marker genes taken from https://metabolicatlas.org/ and constructed the UMAP only on these

genes (Supplementary figure 2D). Performing *Louvain* clustering based on the metabolic

genes resulted in the same number of clusters as when using all genes. Moreover, 97.4% of

the cells were assigned to the same cluster (Supplementary figure 2E). Hence, the same

hepatocyte subgroups are retrieved from this analysis, indicating that indeed metabolic differences are the main driver separating subgroups of hepatocytes.

***New figure in revised manuscript.
Supp. Figure* 2** *Louvain clustering based on the metabolic genes resulted in the same number of clusters as* *when using all genes. Moreover, 97.4% of the cells were assigned to the same cluster.*

**3) Third, do you have any evidence that lipid accumulation (or stimulation) affects drug**

**metabolism and not drug uptake?**

This is a very interesting question, and we have

extended our analysis to thoroughly address it. For that reason, we have analyzed the gene expression of 28 influx transporters in each hepatocyte subgroup and in all the treatments. These transporters include ATP-binding cassette transporters (ABC), which are involved in diverse physiological processes including export of cholesterol, bile salts, and metabolic end products [17]; we have also studied other solute carrier (SLC) transporters that facilitate the transport of a wide array of substrates including nutrients, drugs and other xenobiotics [18]. The new Supplementary Figure 8, show a heatmap depicting log2-fold change to DMSO

level of influx transporter genes in Cocktail and

FFA+Cocktail treatment. We do observe an overall downregulation of the gene expression patterns after FFA and drug cocktail treatment. Several transporters, such as SLCO47A1, SLCO2B1, and ABCG2 are up-

regulated upon cocktail treatment only in the

hepatocyte subgroup II.

These results support the notion that chronic lipid accumulation affect the metabolism of drugs in a subgroup-dependent manner.

***New figure in revised manuscript.***

***Supp. Figure* 8** *Heatmap depicting log2-fold*

*change to DMSO level of influx transporter genes*

*in Cocktail and FFA+Cocktail treatment.*

Bibliography:

1. Scialdone A, Natarajan KN, Saraiva LR, Proserpio V, Teichmann SA, Stegle O, Marioni JC, Buettner F: **Computational assignment of cell-cycle stage from single-cell transcriptome data.** *Methods* 2015, **85:**54-61.
2. Zhang K, Zhang L, Liu W, Ma X, Cen J, Sun Z, Wang C, Feng S, Zhang Z, Yue L, et al: **In Vitro Expansion of Primary Human Hepatocytes with Efficient Liver Repopulation Capacity.** *Cell Stem Cell* 2018, **23:**806-819 e804.
3. Aizarani N, Saviano A, Sagar, Mailly L, Durand S, Herman JS, Pessaux P, Baumert TF, Grün D: **A human liver cell atlas reveals heterogeneity and epithelial progenitors.** *Nature* 2019, **572:**199-204.
4. MacParland SA, Liu JC, Ma X-Z, Innes BT, Bartczak AM, Gage BK, Manuel J, Khuu N, Echeverri J, Linares I, et al: **Single cell RNA sequencing of human liver reveals distinct intrahepatic macrophage populations.** *Nature Communications* 2018, **9:**4383.
5. Stein TA, Burns GP, Tropp BE, Wise L: **Hepatic fat accumulation during liver regeneration.** *Journal of Surgical Research* 1985, **39:**338-343.
6. Zou Y, Bao Q, Kumar S, Hu M, Wang GY, Dai G: **Four waves of hepatocyte proliferation linked with three waves of hepatic fat accumulation during partial hepatectomy-induced liver regeneration.** *PLoS One* 2012, **7:**e30675.
7. Caldez MJ, Bjorklund M, Kaldis P: **Cell cycle regulation in NAFLD: when imbalanced metabolism limits cell division.** *Hepatol Int* 2020, **14:**463-474.
8. Liao Y, Shikapwashya ON, Shteyer E, Dieckgraefe BK, Hruz PW, Rudnick DA: **Delayed Hepatocellular Mitotic Progression and Impaired Liver Regeneration in Early Growth Response-1-deficient Mice*.** *Journal of Biological Chemistry* 2004, **279:**43107-43116.
9. Liu J, Lu YF, Corton JC, Klaassen CD: **Expression of cytochrome P450 isozyme transcripts and activities in human livers.** *Xenobiotica* 2021, **51:**279-286.
10. Scheidecker B, Shinohara M, Sugimoto M, Danoy M, Nishikawa M, Sakai Y: **Induction of in vitro Metabolic Zonation in Primary Hepatocytes Requires Both Near-Physiological Oxygen Concentration and Flux.** *Front Bioeng Biotechnol* 2020, **8:**524.
11. Tonon F, Giobbe GG, Zambon A, Luni C, Gagliano O, Floreani A, Grassi G, Elvassore N: **In vitro metabolic zonation through oxygen gradient on a chip.** *Scientific Reports* 2019, **9:**13557.
12. Wahlicht T, Vièyres G, Bruns SA, Meumann N, Büning H, Hauser H, Schmitz I, Pietschmann T, Wirth D: **Controlled Functional Zonation of Hepatocytes In Vitro by Engineering of Wnt Signaling.** *ACS Synthetic Biology* 2020, **9:**1638-1649.
13. Zhang X, Shen J, Man K, Chu ESH, Yau TO, Sung JCY, Go MYY, Deng J, Lu L, Wong VWS, et al: **CXCL10 plays a key role as an inflammatory mediator and a non­invasive biomarker of non-alcoholic steatohepatitis.** *Journal of Hepatology* 2014, **61:**1365-1375.
14. Kang YB, Eo J, Mert S, Yarmush ML, Usta OB: **Metabolic Patterning on a Chip: Towards in vitro Liver Zonation of Primary Rat and Human Hepatocytes.** *Scientific Reports* 2018, **8:**8951.
15. Danoy M, Poulain S, Lereau-Bernier M, Kato S, Scheidecker B, Kido T, Miyajima A, Sakai Y, Plessy C, Leclerc E: **Characterization of liver zonation-like transcriptomic patterns in HLCs derived from hiPSCs in a microfluidic biochip environment.** *Biotechnology Progress* 2020, **36:**e3013.
16. Ben-Moshe S, Shapira Y, Moor AE, Manco R, Veg T, Bahar Halpern K, Itzkovitz S: **Spatial sorting enables comprehensive characterization of liver zonation.** *Nature metabolism* 2019, **1:**899-911.
17. Vasiliou V, Vasiliou K, Nebert DW: **Human ATP-binding cassette (ABC) transporter family.** *Hum Genomics* 2009, **3:**281-290.

18.	Lin L, Yee SW, Kim RB, Giacomini KM: **SLC transporters as therapeutic targets:**

**emerging opportunities.** *Nat Rev Drug Discov* 2015, **14:**543-560

**Second round of review**

**Reviewer 1**

The authors have made a valid effort to revise their manuscript but the fundamental problem has not changed: this manuscript contains very limited data (based on commercially available human hepatocytes) and there is on overabundance of bioinformatic analysis. Although the latter is interesting, almost none of the findings have been validated by actual experiments. The authors try to compare their data to other existing datasets but this is not the same than doing a real experiment. Overall, it is not clear to this reviewer what should be the contribution of this work. The conclusions are vague and there is a low chance that they will stand the test of time.
Rev1-2: although it is nice the authors acknowledge this issue but they are not doing anything to improve this point. BTW, what does actually "primary human hepatocytes" mean? This may be a simple question but believe me the answer is quite complicated…
Rev1-4: Here the authors used FACS analysis to validate their "cyclone" prediction, which is good. Nevertheless, the percentage G2M (13.9%) is quite different from Cyclone (probably 2%). Does this mean that the Cyclone prediction is not very accurate?
Rev2-3: the reviewer asks an important question but instead of doing the right experiments, the authors do more bioinformatics. As a result, the data becomes very indirect and is hard to interpret.
At the beginning of the Result section, it is mentioned that "average of 2,550 genes per cell" were detected. This is around 15-20% coverage of the genome. Of course, this is always a problem with single cell sequencing but also a major weakness of this manuscript.

**Reviewer 2**

The revision addressed my comments.

It was reassuring to see that the results of analysis (clusters etc.) did not change after reducing the genes to metabolic genes only. This provides a strong evidence supporting the finding that metabolism is the strongest driver for the observed hepatocytes grouping.

**Authors Response**

**Point-by-point responses to the reviewers’ comments:**

**Reviewer #1:** The authors have made a valid effort to revise their manuscript but the fundamental problem has not changed: this manuscript contains very limited data (based on commercially available human hepatocytes) and there is on overabundance of bioinformatic analysis. Although the latter is interesting, almost none of the findings have been validated by actual experiments. The authors try to compare their data to other existing datasets but this is not the same than doing a real experiment. Overall, it is not clear to this reviewer what should be the contribution of this work. The conclusions are vague and there is a low chance that they will stand the test of time.

**Rev1-2:** although it is nice the authors acknowledge this issue but they are not doing anything to improve this point. BTW, what does actually "primary human hepatocytes" mean? This may be a simple question but believe me the answer is quite complicated...

Primary human hepatocytes are defined as hepatocytes isolated from liver and maintained in culture for a few days, where they retain their adult phenotype [1]. The cells purchased in this manuscript (Lonza), have the same definition: “Primary Cells – Cells isolated directly from human or animal tissue using enzymatic or mechanical methods. Once isolated, they are placed in an artificial environment in plastic or glass containers supported with specialized medium containing essential nutrients and growth factors to support proliferation.” Hepatocytes were isolated using a two-step collagenase perfusion protocol as described by the manufacturer, which is the standard method to isolate hepatocytes from livers, in mouse and humans [2-6]

.

**Rev1-4:** Here the authors used FACS analysis to validate their "cyclone" prediction, which is good. Nevertheless, the percentage G2M (13.9%) is quite different from Cyclone (probably 2%). Does this mean that the Cyclone prediction is not very accurate?

As the reviewer mentioned, FACS and Cyclone analysis are two different cell cycle analysis methods. We agree that the computational prediction of cell cycle has it challenges, and some methods might be suitable for a specific cell type or treatment/condition [7]. We have selected Cyclone [8] because it was developed and validated using hepatocytes, and because its results are consistent with other inferring methods. For instance, we have performed an unbiased scoring analysis using 1263 unique G1 marker genes listed in [cyclebase.org](http://cyclebase.org) [9]. Then, using the Scanpy pipeline [10] and its internal gene scoring function, we scored our hepatocytes based on those G1 marker genes. As expected, most of the hepatocytes were found in G1/G0 cell cycle stage as predicted by Cyclone.

Experimentally, cell cycle analysis by FACS relies on dyes such as Hoechst [11], and 4n hepatocytes (or 4c hepatocytes) are identified in G2/M phase [12] (schematic representation in Figure 1 for Reviewer A). However, Cyclone uses the expression of cell cycle marker genes, and diploid and tetraploid hepatocytes are both identified in the G0/G1 phase as quiescent cells (schematic representation in Figure 1 for Reviewers D). Therefore, the main differencesin the percentage of G2/M observed between FACS and Cyclone are based on the terminology and the methodology to identify the two different cellular states of hepatocytes (diploid and tetraploid hepatocytes) (Figure 1 for Reviewers C).

To support this, we have selected cell cycle marker genes such as *DYNLL1* for G1 phase, *ARGLU1* for S phase, and *SMCA* for G2/M phase (Figure 1 for Reviewer E). As expected, most of the hepatocytes remain in G1/G0 phase. To monitor the percentage of diploid and tetraploid hepatocytes, we have calculated the number of cells expressing three or more polyploid marker genes [13]. In Figure 1 for Reviewer F, we can see that most of the hepatocytes co-expressing ≥3 polyploid marker genes (14.14% green) are in G1/G0 cell state, and a total of 19% of all cells co-expressing at least 3 markers of polyploidy also matches the 17% of G2/M cells identified by FACS analysis. These results suggest, as expected, that diploid and polyploid hepatocytes are both identified in G1/G0 phase using Cyclone. This percentage of polyploid hepatocytes also matches the percentage of tetraploid hepatocytes described for human livers [14].

**Figure for Reviewers 1.** Comparison between cell cycle analysis using FACS and Cyclone. A) Scoring of cells based on expression of G1 marker genes shows comparable results to Cyclone analysis. B) Illustrative representation (cartoon) of tetraploid hepatocytes identified in G2/M phase using FACS analysis and Hoechst. C) Percentage of Cell cycle estimations between FACS and Cyclone analysis. D) Illustrative representation (cartoon) of Cyclone and cell cycle markers genes identifying diploid and tetraploid hepatocytes in G1/G0. E) Dot plot of cell cycle marker genes showing that primary hepatocytes are mainly in G1/G0 phase as expected. F) Percentage of hepatocytes with 3 or more polyploid marker genes co-expressed in different cell cycle stages showing that potential tetraploid hepatocytes are capture in G1 phase using Cyclone.

**Bibliography**

1. Castell JV, Jover R, Martnez-Jimnez CP, Gmez-Lechn MJ: **Hepatocyte cell lines: their use, scope and limitations in drug metabolism studies.** *Expert Opinion on Drug Metabolism & Toxicology* 2006, **2:**183-212.
2. Lee SM, Schelcher C, Demmel M, Hauner M, Thasler WE: **Isolation of human hepatocytes by a two-step collagenase perfusion procedure.** *J Vis Exp* 2013.
3. Charni-Natan M, Goldstein I: **Protocol for Primary Mouse Hepatocyte Isolation.** *STAR Protoc* 2020, **1:**100086.
4. Feng M, Divall S, Wu S: **An Improved Time- and Labor- Efficient Protocol for Mouse Primary Hepatocyte Isolation.** *J Vis Exp* 2021.
5. G0mez-Lech0n MJ, Donato MT, Castell JV, Jover R: **Human hepatocytes in primary culture: the choice to investigate drug metabolism in man.** *Curr Drug Metab* 2004, **5:**443-462.
6. G0mez-Lech0n MJ, Donato MT, Castell JV, Jover R: **Human hepatocytes as a tool for studying toxicity and drug metabolism.** *Curr Drug Metab* 2003, **4:**292-312.
7. Hsiao CJ, Tung P, Blischak JD, Burnett JE, Barr KA, Dey KK, Stephens M, Gilad Y: **Characterizing and inferring quantitative cell cycle phase in single-cell RNA-seq data analysis.** *Genome Res* 2020, **30:**611-621.
8. Scialdone A, Natarajan KN, Saraiva LR, Proserpio V, Teichmann SA, Stegle O, Marioni JC, Buettner F: **Computational assignment of cell-cycle stage from single-cell transcriptome data.** *Methods* 2015, **85:**54-61.
9. Gauthier NP, Jensen LJ, Wernersson R, Brunak S, Jensen TS: [**Cyclebase.org:**](http://Cyclebase.org:) **version 2.0, an updated comprehensive, multi-species repository of cell cycle experiments and derived analysis results.** *Nucleic Acids Res* 2010, **38:**D699-702.
10. Wolf FA, Angerer P, Theis FJ: **SCANPY: large-scale single-cell gene expression data analysis.** *Genome Biol* 2018, **19:**15.
11. Belloc F, Dumain P, Boisseau MR, Jalloustre C, Reiffers J, Bernard P, Lacombe F: **A flow cytometric method using Hoechst 33342 and propidium iodide for simultaneous cell cycle analysis and apoptosis determination in unfixed cells.** *Cytometry* 1994, **17:**59-65.
12. Duncan AW, Taylor MH, Hickey RD, Hanlon Newell AE, Lenzi ML, Olson SB, Finegold MJ, Grompe M: **The ploidy conveyor of mature hepatocytes as a source of genetic variation.** *Nature* 2010, **467:**707-710.
13. Donne R, Sangouard F, Celton-Morizur S, Desdouets C: **Hepatocyte Polyploidy: Driver or Gatekeeper of Chronic Liver Diseases.** *Cancers (Basel)* 2021, **13**.
14. Wang MJ, Chen F, Lau JTY, Hu YP: **Hepatocyte polyploidization and its association with pathophysiological processes.** *Cell Death Dis* 2017, **8:**e2805.
